# Engineering microbial pathways for production of bio-based chemicals from lignocellulosic sugars: current status and perspectives

**DOI:** 10.1186/s13068-020-01744-6

**Published:** 2020-07-08

**Authors:** Jean Marie Francois, Ceren Alkim, Nicolas Morin

**Affiliations:** 1 Toulouse Biotechnology Institute, CNRS, INRA, LISBP INSA, 135 Avenue de Rangueil, Toulouse Cedex 04, 31077 France; 2 Toulouse White Biotechnology (TWB, UMS INRA/INSA/CNRS), NAPA CENTER Bât B, 3 Rue Ariane 31520, Ramonville Saint-Agnes, France

**Keywords:** White biotechnology, Lignocellulose, Microbial physiology, Metabolic engineering, Synthetic biology, Bio-based products, Chemicals

## Abstract

Lignocellulose is the most abundant biomass on earth with an annual production of about 2 × 10^11^ tons. It is an inedible renewable carbonaceous resource that is very rich in pentose and hexose sugars. The ability of microorganisms to use lignocellulosic sugars can be exploited for the production of biofuels and chemicals, and their concurrent biotechnological processes could advantageously replace petrochemicals’ processes in a medium to long term, sustaining the emerging of a new economy based on bio-based products from renewable carbon sources. One of the major issues to reach this objective is to rewire the microbial metabolism to optimally configure conversion of these lignocellulosic-derived sugars into bio-based products in a sustainable and competitive manner. Systems’ metabolic engineering encompassing synthetic biology and evolutionary engineering appears to be the most promising scientific and technological approaches to meet this challenge. In this review, we examine the most recent advances and strategies to redesign natural and to implement non-natural pathways in microbial metabolic framework for the assimilation and conversion of pentose and hexose sugars derived from lignocellulosic material into industrial relevant chemical compounds leading to maximal yield, titer and productivity. These include glycolic, glutaric, mesaconic and 3,4-dihydroxybutyric acid as organic acids, monoethylene glycol, 1,4-butanediol and 1,2,4-butanetriol, as alcohols. We also discuss the big challenges that still remain to enable microbial processes to become industrially attractive and economically profitable.

## Background

**Fig. 1:**
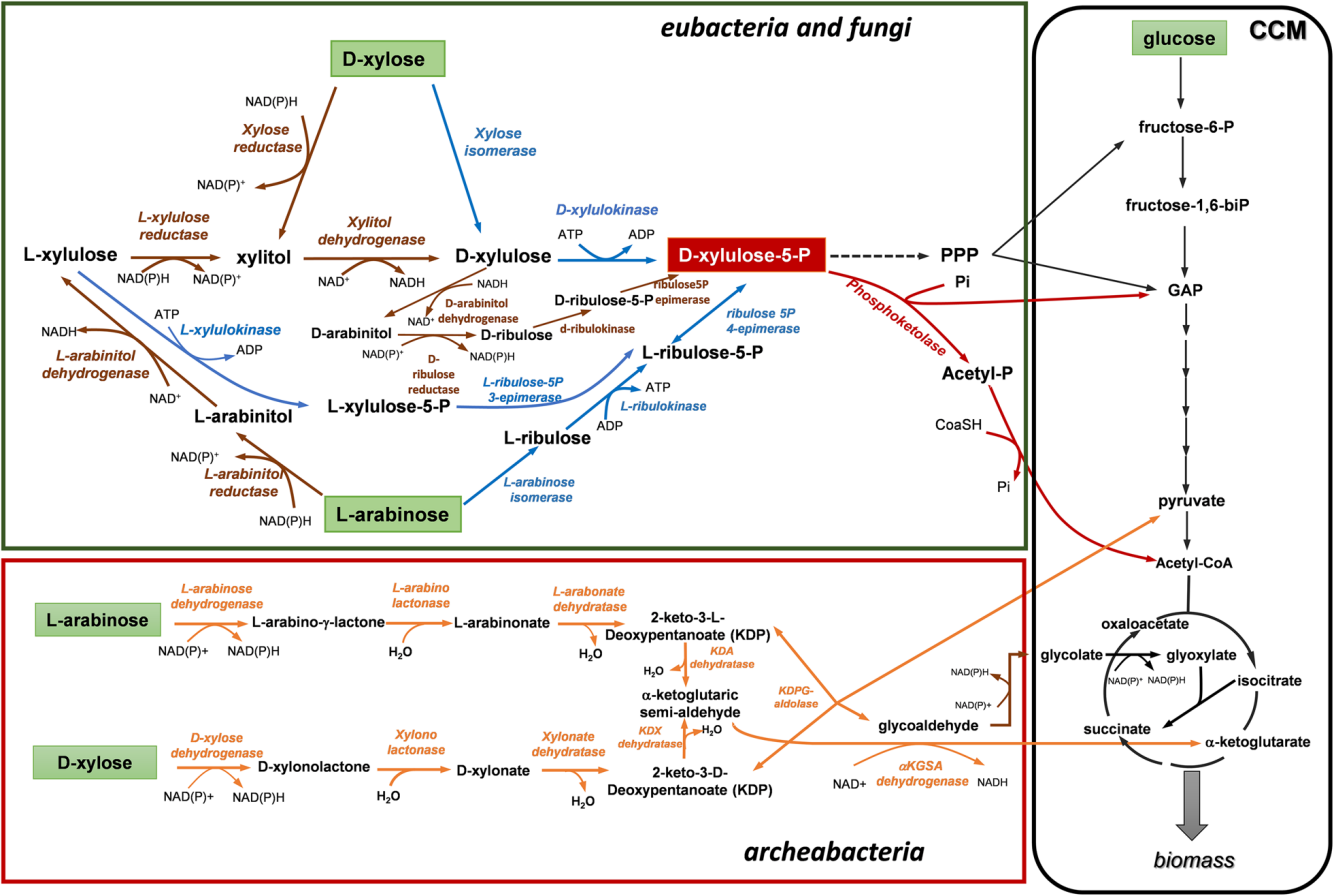
Overview of the metabolic pathways, which enables fungi, eubacteria and Archea to assimilate pentose and hexose sugars from growth and biomass. Brown, blue and orange arrows and letters mean the pathways used by fungi, eubacteria and Archea, respectively. The phosphoketolase reaction is present in bacteria and fungi and is represented by red arrows and letters. CCM: Carbon central metabolism

The 21st century is challenging the human society with major social and economic issues owing to the irreversible global warming and the steady rise of environmental pollution, both events mainly resulting from our slave reliance on fossil resources. The expected rarefaction of these resources prompted us to innovate in alternative and sustainable solutions, which in particular must revive the natural cycles of carbon and nitrogen. One of the cornerstones of this new societal and economical deal coined ‘Bioeconomy’ [1, 2] stands on our ability to exploit renewable carbon resources to produce environmentally gentle fuels and chemicals that shall profitably replace those derived at present from fossil resources. Among the potential renewable resources, lignocellulosic biomass is the most abundant feedstock on earth with an annual output of around 2 × 10^11^ metric tons [3]. This feedstock is a non-edible carbon source, which enables to circumvent the resource contention for ‘food’ versus ‘chemicals and fuels’ purposes [4]. In addition to cellulose, which is a polymer of β-1, 4 linked glucosyl units, lignocellulose is composed of a hemicellulosic fraction that can reach 75% of total biomass whose major sugars are d-xylose and l-arabinose [5]. Microorganisms are naturally able to grow on pentose sugars either through the oxidative pentose phosphate pathway (PPP) for Eubacteria [6] and fungi [7] or via the pentose oxidation pathway in Archea [8–10] (Fig. 1). However, with the desire to fully convert lignocellulosic sugars into bio-based natural or non-natural products, the classical metabolic network harbored by these microorganisms is not optimally shaped for this purpose. Synthetic biology and evolutionary engineering emerge as very promising scientific and technological strategies that can meet the biotechnological objective without disrupting cellular homeostasis and keeping at minimum growth of microbial system. Applied to microbial organism, synthetic biology consists in rewiring existing pathways with novel enzymatic functions and/or to implement new non-natural-metabolic pathways in a microbial chassis with the ensuing goal of maximizing product yield, titer and productivity [11, 12]. In this review, we will first recall the existing natural pathways that have been elaborated by microbial organisms to assimilate pentose and hexose (coined C5 and C6 sugars). We will then report on the exploitation of the non-phosphorylating pentose pathway, the reconstruction and implementation of natural and non-natural pathways into industrially tractable microorganisms, enabling efficient conversion of lignocellulosic sugars into a wide range of economically attractive bio-based chemicals. Biofuels and bioethanol are not considered in this review as these bio-based products have received a lot of attention and reviews over the last 10 years [13–17]. In addition, this review complements and extends that of [18], which was focused on metabolic engineering strategies for the production of C2–C4 diols from lignocellulosic sugars and that of [19], which described specific advancements on engineering strategies for utilization and conversion of xylose into chemicals. We will conclude about the challenges that remain to incorporate these microbial cell factories as key players in the emerging Bio-based Economy.

## Overview of the natural metabolic pathways for microbial assimilation of lignocellulosic sugars

Pentose and hexose sugars derived from lignocellulosic biomass are ideal carbon source for the production of bio-platform molecules by industrially tractable microorganisms such as *E. coli, Corynebacterium glutamicum* and yeasts. However, a major limiting factor in the use of these sugars is the difficulty to extract them from lignocellulosic material due to the extreme structural complexity of this feedstock. Though it is not the purpose here to discuss about lignocellulose features, which has been treated in many excellent reviews [20–22], it is worth to notice that deconstruction and solubilization of lignocellulose are strategic issues for commercialization of biomass-based technologies. Relevant progress in this field has been recently reviewed, notably in three directions which are actually complementary. A first one is the recent discovery of deep eutectic solvents (DESs), also termed green solvents showing high-efficient biomass deconstruction and being more compatible for future treatment with enzymes and microorganisms [23]. A second direction is focusing on engineering the composition and structure of the cell wall with the ultimate goal to create tailor-made biomass with a cell wall optimized for particular industrial or agricultural applications, without affecting plant growth and development [24, 25]. Innovation in this research field would also come from engineering native biomass biograders, such as *Clostridium thermocellum* or *Caldicellulosiruptor* [26, 27] that are able to convert directly lignocellulose into monomers and thus could fulfill the consolidated bioprocessing concept originally set up by Lynd et al. in which biomass conversion into products can be carried out by a single fermenting microbe [28].

Prokaryotic and eukaryotic microorganisms have elaborated different pathways to assimilate pentose (d-xylose and l-arabinose) and hexose (d-glucose) sugars as depicted in Fig. 1. Table 1 provides a summary of genes and their corresponding encoded enzymes involved in pentose assimilation pathways by bacteria and fungi. When data are available, catalytic efficiencies of these enzymes on their specific substrates are also reported. In filamentous fungi and xylose-fermenting yeasts, d-xylose and l-arabinose are metabolized by an oxidoreductive pathway (Fig. 1, arrows and symbols in brown) into xylulose-5-P (Xu5P), which thereafter enters the central carbon metabolism (CCM) via the non-oxidative branch of the pentose phosphate pathway (PPP). The assimilation of these pentoses begins with their uptake that is carried out by a relatively large, yet poorly characterized sugar transporter family [29–31] (not illustrated in the picture). They are reduced into their corresponding alcohol by an NADPH-dependent d-xylose (or l-arabinose) reductase, followed by a dehydrogenation catalyzed by an NAD^+^-dependent xylitol (or l-arabitol) dehydrogenase to yield d-xylulose and l-xylulose, respectively. d-Xylulose is readily phosphorylated on carbon 5 by a d-xylulokinase to d-xylulose-5-P, which fuels the CCM at the level of fructose-6-P and glyceraldehyde-3-phosphate (GAP) through transketolase/transaldolase-catalyzed reactions. Prior to its incorporation into CCM, l-xylulose derived from l-arabinose must be reduced into xylitol by an NADPH-dependent l-xylulose reductase followed by an oxidation catalyzed by an NAD^+^-xylitol dehydrogenase [32]. In some fungi, such as *Pichia stipitis*, d-xylulose can enter the non-oxidative pentose phosphate pathway via another route, which involves its reduction into d-arabinitol, followed by oxidation to d-ribulose and then phosphorylation to ribulose-5-P [33]. Overall, the assimilation of d-xylose and l-arabinose by filamentous fungi is characterized by a dissimilarity in the use of redox cofactors while the process is redox neutral. This dissimilarity of redox cofactors is still considered as a major problem for efficient fermentation of pentose sugars into ethanol by metabolically engineered yeasts [34].

**Table 1:** Description of enzymes and corresponding genes implicated in microbial assimilation of lignocellulosic sugars

Pathway	Enzyme name	Enzyme abbreviation	Gene name	Specific constant kcat/Km (s^−1^ M^−1^) [substrat]	Microbial origin	Reference
Pentose oxido-reductive pathway	d-Xylose reductase	XR	*XYL1* xyrA	na na	*P. stipitis* *A. niger*	[32]
	Xylitol dehydrogenase	XDH	xdh1 *XYL2*	na na	*Filamentous fungi* *S. cerevisiae*	[136] [137]
	d-Xylulokinase	XK	xylB	879 10^3^ [xylulose]	*E. coli*	[138]
			*XKS1* *xkiA* *XYL3*	na	*S. cerevisiae* *Aspergillus niger* *Pichia stipitis*	[139] [140] [141]
	l-Arabinose reductase	AR	*XYL1* *larA*	na na	*T. reesei* *Aspergilus niger*	[32] [142]
	l-Arabitol 4-dehydrogenase	ARDH	ladA	na	Filamentous fungi*	[33, 143]
	l-Xylulose reductase (NADPH) l-Xylulose reductase (NADH)	lXR	*lrx3* *lxrA* *ALX1*	712 [l-xylulose] 47,10^3^ [l-xylulose] na	*T. reesei* *Aspergillus niger* *Ambrosiozyma monospora*	[144] [145] [146]
	d-Arabinitol 2-dehydrogenase/ribulose reductase	RIR	*ARD*	na	*Pichia stipitis* *Candida tropicalis*	[33] [147]
	d-Ribulokinase	RIK	*YDR109c*	80 10^3^ [D-ribulose]	*S.cerevisiae,*	[148]
Isomerase pathway	Xylose isomerase	XI	xylA	na	*E. coli*	[149]
	d-Xylulokinase	XK	xylB	879 10^3^ [xylulose]	*E. coli*	[138]
	l-Arabinose isomerasz	AI	araA	na	*E. coli*	[150]
	l-Ribulokinase	RKI	araB	na	*E. coli*	[151, 152]
	l-Xylulokinase	XKI	xylK	na	*E. coli*	[108]
	l-Ribulose-5-P 3-epimerase	UlaE	*ulaE*	na	*E. coli*	[153]
	l-Ribulose 5-P 4-epimerase	RPE	araD	na	*E. coli*	[154]
Non-phosphorylative pathway	d-Xylose dehydrogenase	XylDH	xylB xylB HVO_B0028	10^4^ [D-Xylose] 157,10^3^ [xylose]	*Caulobacter crescenti* *S.solfataricus* *H. vulcanii*	[46, 48, 155, 156]
	d-Xylonate lactonase	XAL	xylC	na na	*C.crescenti* *H.volcanii*	[48, 81]
	d-Xylonate dehydratase	XAD	xylD HVO B0038A yagE/yjhG	40 10^3^ [D-xylonate) 4 10^3^ (D-Xylonate)	*C. crescenti* *H.volcanii* *Escherichia coli*	[48, 70, 155]
	l-Arabinose 1-dehydrogenase	AraDH	araA	32.3 10^3^[l-arabinose]	*B. multivorans*	[81]
	l-Arabinolactonase	AraL	araL	na	*B. multivorans,* *S. solfataricus*	[81]
	l-Arabinonate dehydratase	AraD	araC	83 [l-arabinonate]	*B.multivorans* *S. solfataricus*	[81] [156]
	2-Keto-3-deoxy-d-xylonate dehydratase	KdxD	xylX	260 [2-keto-3-deoxy-D-pentanoate] 530 [2-keto-3-deoxy-D-pentanoate]	*C. crescenti* *B. xenovorans*	[80, 81]
	2-Keto-3-deoxy-l-arabinonate dehydratase	KdaD	araD	23 [2-keto-3-deoxy-l-arabinonate]	*B. xenovorans*	[81]
	2-Ketoglutarate semialdehyde dehydrogenase	KGSADH	xylA ycbD HV0_B0039	4.6 10^5^ [α-ketoglutaric semialdehyde]	*A. brasilense* *C. crescenti* *S. solfataricus* *H. valcanii*	[48, 155–157]
	2-Keto-3-deoxy-D/l-pentanoate aldolase	KDPA	kdxA yagE/yjhH	450 [2-keto-3-deoxy-D-pentanoate] 7000 [pyruvate]	*S. solfataricus* *Escherichia coli*	[47, 62]

na: Not accessible

In eubacteria (e.g., *Escherichia coli*, *Corynebacterium*, *lactococcus, Streptomyces* species, etc.), the assimilation of pentose sugars also starts with their uptake mediated by active transporters [29]. Once they get into the cell, they are isomerized by a corresponding isomerase into d-xylulose or l-ribulose. d-xylulose further enters the CCM via Xu5P, by direct phosphorylation catalyzed by a d-xylulokinase. Alternatively Xu5P can be obtained from l-ribulose through a phosphorylation and epimerization catalyzed by a l-ribulokinase and a l-ribulose 5-P 4-epimerase, respectively (Fig. 1, arrows in blue). The isomerase pathway is thus making the distinction between eubacteria and fungi for pentose assimilation as the latter relies on an oxido-reductive pathway. Nevertheless, exceptions exist such as for the anaerobic fungi *Pyromyces* sp. that can metabolize d-xylose via the isomerase route [35], whereas the bacteria *Mycobacterium smegmatis* utilizes the oxido-reductive pathway for assimilation of d-arabinose [36]. Another deviation to this general rule of pentose assimilation is the phosphorolytic cleavage of Xu5P into glyceraldehyde-3P (GAP) and acetyl-Pi catalyzed by a xylulose-5-P/fructose-6-P phosphoketolase (PK) (Fig. 1, arrows in red). This PK route has been originality identified in lactic bacteria and bifidobacteria [37–40], and turns out to be widely present in filamentous fungi [41] and unicellular algae [42, 43]. In most cases, PPP and PK routes coexist in a same organism, raising the question about which is the most useful for pentose assimilation. In *Clostridium acetobutylicum* for which this question has been recently addressed it, was found that the PK route is more useful than PPP and that l-arabinose is assimilated before d-xylose [40].

In a third pathway, which is the privilege of archeabacteria (Fig. 1, arrows and symbols in orange), d-xylose and l-arabinose (as well as d-ribose or d-arabinose) are catabolized by a so-called non-phosphorylating pentose (NP) pathway to either α-2-ketoglutarate (αKG) or pyruvate and glycoladehyde. The conversion of pentose into αKG takes place by the Weimberg pathway [8]. This pathway proceeds in five consecutive enzymatic steps. The first step is the oxidation of the pentose (d-xylose or l-arabinose) into pentono-(d/l)-lactone (d-xylonolactone or l-arabinolactone) by a specific pentose dehydrogenase (i.e., d-xylose dehydrogenase or l-arabinose dehydrogenase). These intermediates are then hydrated by a specific lactonase into their corresponding pentonic acid, followed by a dehydration into a 2-keto-3-deoxy l or d-pentanoate (KDP) catalyzed by specific d-xylonate or l-arabinonate dehydratase. A subsequent dehydratation catalyzed by a 2-keto-deoxy l- or d-pentanoate dehydratase (KDPH) gives rise to α-ketoglutaric semialdehyde also termed 2,5-dioxopentanoate (DOP). The last reaction of this pathway is catalyzed by an α-ketoglutaric semialdehyde dehydrogenase that yields α-ketoglutarate, which fuels CCM at the level of the tricarboxylic acid cycle (TCA) [9, 44]. An alternative route to this catabolic pathway, which has been discovered by Dahms [45] and is therefore termed the ‘Dahms pathway’, is the aldolytic cleavage of KDP into pyruvate and glycolaldehyde by a KDP aldolase. While pyruvate directly fuels the CCM, glycolaldehyde is incorporated into CCM at the level of glyoxylate, which requires two reactions generating two reduced equivalents. This was demonstrated in *Sulfolobus* sp., engaging a highly oxygen-sensitive glycoladehyde oxidoreductase and a soluble NADH-dependent glyoxylate reductase [46]. Although the NP pathway has been known for over 50 years, there are still many issues to be clarified regarding the promiscuity of enzymes in this pathway, the dependence to redox cofactors NAD^+^ or NADP^+^ as well as the co-existence of the Weimberg and Dahms route in a same microorganism. In this respect, Nunn et al. [46] showed that the hyperthermophilic archea *Sulfobolus solfataricus* and *acidocaldarius* employ the same enzymes for the oxidation of d-xylose and l-arabinose to generate 2-keto-3-deoxy-d-xylonate (KDX) and 2-keto-3-deoxy-l-arabinonate (kDa), which thereafter are cleaved by a same KDP aldolase into pyruvate and glyceraldehyde [47]. In these archeabacteria, the Weimberg and Dahms route coexists at a ratio of about 50/50 [46], whereas in other bacteria such as *Haloferax volcanii*, d-xylose is mainly metabolized by the Weimberg pathway and the xylose dehydrogenase has almost no activity on other C5 sugars [48].

## The non-phosphorylating pentose pathway combined with the central carbon metabolism for conversion of lignocellulosic sugars into bio-based chemicals

### Main reason to refactor carbon metabolism for biotechnological objectives

As stated above, many microorganisms are able to assimilate pentose and hexose sugars. However, this capability is not economically viable with the biotech industry sector aiming at producing bio-based chemicals from these lignocellulosic sugars, due to several constraints as follows. First, pentose assimilation is often repressed in the presence of glucose [49, 50]. This can strongly hampers the fermentation process, and different strategies have been elaborated to alleviate this glucose repression effect. These include engineering hexose transporter to release xylose uptake from glucose inhibition in yeast [51], abolition of the cAMP-CRP-dependent glucose repression in bacteria [52], adapted laboratory evolution [53–55], use of a microconsortium of different *E. coli* strains, each engineered to preferentially catabolize a different hexose–glucose, galactose, or mannose [56], as well as fermentation strategy such as continuous culture [57]. Second, the common metabolite intermediate from C5 and C6 metabolism is a C3 compound, such as GAP or PEP, which can be problematic if the desired chemical is made with C4 or C5 carbon skeleton. Yet importantly, traditional pathways comprise lengthy enzymatic reactions that are subject to transcriptional and posttranscriptional regulation, whose complexity hampers performance in terms of yield and productivity.

**Fig. 2:**
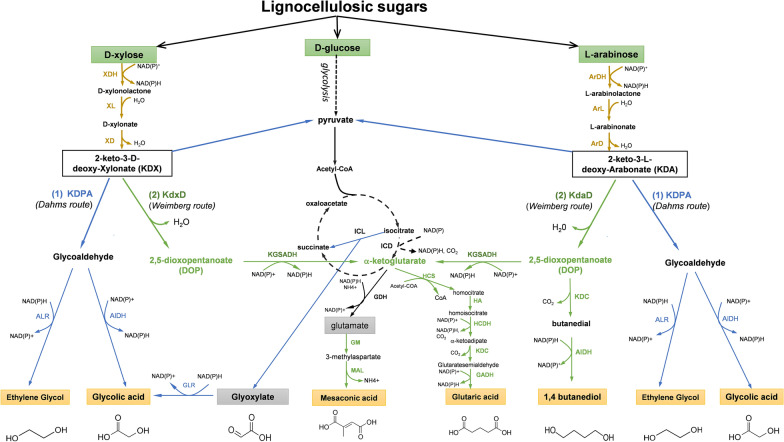
Link between the Dahms (pathway [1]) or Weimberg-dependent (Pathway [2]) non-phosphorylative pathway to central carbon metabolism (CCM). XDH: xylose dehydrogenase; XL: xylulolactonase; XD: xylonate dehydratase; KDPA: 2-keto-3-deoxy-pentanoate aldolase; KdxD: 2-keto-3-deoxy-d-xylonate dehydratase, KdaD: 2-keto-3-deoxy-l-arabonate dehydratase; KGSADH: a ketoglutarate semialdehyde dehydrogenase; ALR: aldehyde reductase; AlDH: aldehyde dehydrogenase; GLR: glyoxylase reductase; GDH: glutamate dehydrogenase; GM: glutamate mutase: MAL: methylaspartate lyase; HCS: homocitrate synthase; HA: homocitrate aconitase; HCDH; homocitrate dehydrogenase; KDC: a-ketoacid decarboxylase; GADH: glutaric acid dehydrogenase. Further details of the enzymes and their encoding genes are given in Additional file 1: Table S1

**Fig. 3:**
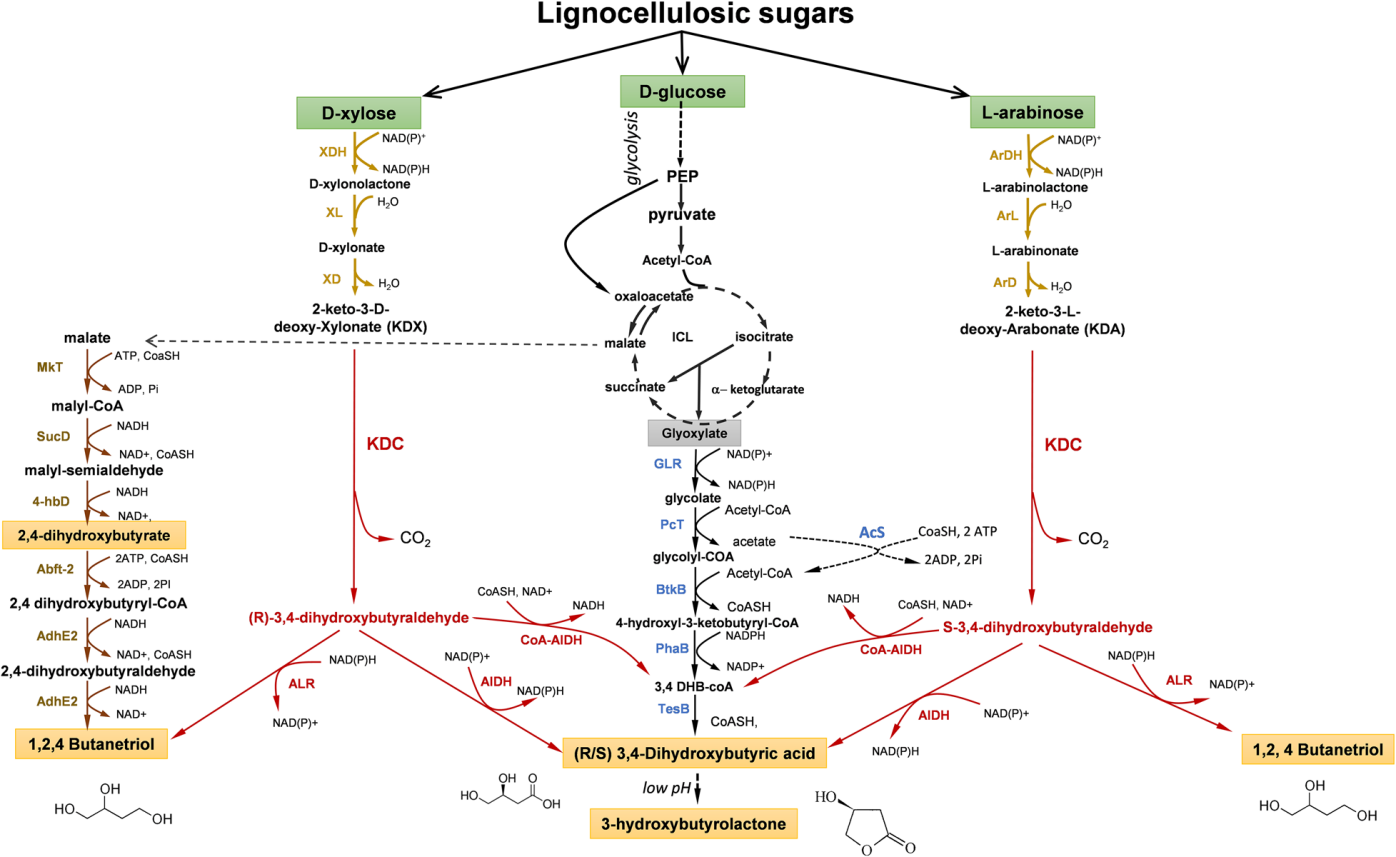
Link of KDC-dependent non-phosphorylative pathway to central carbon metabolism (CCM). XDH: xylose dehydrogenase; XL: xylulolactonase; XD: xylonate dehydratase; KDC: a-ketoacid decarboxylase; ALR: aldehyde reductase; AlDH: aldehyde dehydrogenase; CoA-AlDH; coenzyme A-acylating aldehyde dehydrogenase; GLR: glyoxylase reductase; PcT: propionyl-CoA transfease; BtkB: butyryl Coa Transferase; PhaB: 3-hydroxybutyryl-CoA reductase; TesB: thioesterase II; MKT: malyl-CoA synthetase, SucD: succinyl-COA reductase; 4hbD: 4 hydroxybutyrate dehydrogenase; Abft-2: 4-hydrobutyrate CoA transferase; AdhE2: bifunctional alaldehyde/alcohol dehydrogenase. Additional details on enzymes and encoding genes are given in Additional file 1: Table S1

These limitations could be also overcome by a rewiring of the existing carbon metabolism pathways to reach the targets in less reaction steps, or by implementing non-natural or artificial pathways that are orthogonal to the classical pathways. The implementation of the nonphosphorylating pentose (NP) pathway present in archeabacteria [44] in industrially relevant microorganisms such as *E. coli*, yeast or *C. glutamicum* [58–60] is an interesting solution, as it can equip the microbial cells with new functionalities to co-assimilate C5 with C6 sugars. The key intermediate of this pathway is 2-keto-3-deoxy-d-xylonate (KDX) or 2-keto-3-l-arabinonate (KDA) obtained from d-xylose or l-arabinose through the three reaction steps involving a dehydrogenase, a lactonase and a dehydratase (Figs. 2 and 3). It should be noted that lactone intermediate can be spontaneously hydrolyzed into d-xylonate and l-arabinonate. This may explain that the genes *xylC* or *araB* encoding these reactions are often not considered in the pathway construction. The genes encoding these enzymes have been isolated from various archaebacteria such as *Caulobacter crescentus* or *Burkholderia multivorans* and heterologously expressed in *E. coli,* yeast or *C. glutamicum* (see Additional file 1: Table S1 for a description of these enzymes, their catalytic efficiency and corresponding genes used in heterologous expression). Also, *E. coli* possesses two genes *yagF* and *yjhG* encoding a dehydratase that catalyzes the dehydration of d-xylonate into KDX [58, 61]. As indicated in Figs. 2 and 3, the produced KDX or KDA can be taken in charge by three potential downstream pathways, to yield a wide range of bio-based products. For simplification, these three pathways have been termed NP_Dahms, NP_Weimberg and NP_KDC. The orthogonality of these pathways enable them to function alone or in connection with the CCM, with the latter being able to provide either growth support or additional product yield, as it will discussed in the next paragraphs.

The bio-based chemicals that are considered at length in this review as they were reported to be produced through these NP pathways are listed in Table 2. These products are nowadays exclusively manufactured by chemical means but their bioproduction from lignocellulose is both scientifically sound and industrially feasible. These chemicals furthermore cover a large spectrum of application in chemical, pharmaceutical and food industries, with a global market size of USD 8.8 billions in 2017 and an expected compound annual growth rate (CAGR) of 12.6% from 2018 to 2025 (see https://www.grandviewresearch.com/industry-analysis/bio-based-platform-chemicals-market).

**Table 2:** Relevant commodity and fine chemicals made from petrochemistry, which are foreseen to be produced by economically viable fermentation process economically from lignocellulosic sugars

Chemicals name	Chemical formula	Molecular mass	Degree of reduction	Application field	Market size (CAGR*) (US dollars)	Reference
Glycolic acid	C_2_H_4_O_3_	76	6	Dyeing and tanning agent in textile industry, flavor and preservative in food industry, skin care agent in cosmetics	280 million by 2019 CAGR 11.3%	https://www.marketsandmarkets.com/Market-Reports/glycolic-polyglycolic-acid-market-1090.html
Mono ethylene glycol	C_2_H_6_O_2_	62	10	Antifreeze agent, precursor of polyethylene terephthalate (PET) for plastic bottle, use in paints and resins in chemical industry,	26 billion by 2018 CAGR 5%	https://www.marketresearchfuture.com/reports/mono-ethylene–glycol-market-2709
Glyoxylic acid	C_2_H_2_O_3_	74	6	Cosmetic, personal care and dermatology, household, adhesive, additive for ink and painting, component in emulsion polymers	76million in 2016 CAGR 5.27%	https://www.marketresearchfuture.com/reports/glyoxylic-acid-market-3158
1,4 Butanediol	C_4_H_10_O_2_	90	22	Fibers, plastics, medicines, cosmfibreetics, artificial leather, pesticides, plasticizers, hardener, solvent, rust remover, etc.	7.75 Billion by 2017 CAGR 6.6%	https://www.marketsandmarkets.com/Market-Reports/1-4-butanediol-market-685.html
1,2,4 Butanetriol	C_4_H_10_O_3_	106	20	Building block for energetic plasticizer, polymers, for electronics materials	8,9 billion by 2025 CAGR 8.2%	https://www.grandviewresearch.com/press-release/global-1-4-butanediol-market
3,4 dihydroxybutyric acid hydroxybutyrolactone	C_4_H_8_0_4_	120	16	Intermediates for high-value pharmaceutical compounds such as Crestor, Lipitor, Zyvox, Zetia	54 million (2003) CAGR 5.5%	http://www.mynewsdesk.com/us/tag/3-hydroxybutyrolactone-market
Glutaric acid	C_5_H_8_O_4_	132	20	Building block for polyesters and polyamides like nylon, ingredient in cleaning products, painting and coating formulation	Data not found	NA
Mesaconic acid	C_5_H_6_0_4_	130	18	Precursor for pentadiene (isoprene) by thermocatalysis	Data not found	NA

*CAGR: compound annual growth rate; NA: not available

### The NP_Dahms metabolic route for ethylene glycol and glycolic acid

The NP_Dahms pathway starts by the aldolytic cleavage of KDX or KDA into glycolaldehyde and pyruvate catalyzed by a 2-keto-3-deoxy-pentanoate aldolase (KDPA). While pyruvate can be used as a carbon source for growth, glycoaldehyde is either oxidized into glycolic acid (GA) or reduced into ethylene glycol (EG) (Fig. 2). In *E. coli*, two genes *yjhH* and *yagE* encode a functional KDPA [62], as evidenced by the finding that a double *yjhH yagE* mutant was unable to grow on d-xylonate [63]. In addition, KDPA encoded by *yagE* is likely more active than that encoded by *yjhH* since levels of GA were higher in engineered strains with *yagE* [63]. The fate of glycolaldehyde to either EG or GA will depend on the expression levels of genes encoding aldehyde reductase or aldehyde dehydrogenase. Oxidation of glycoladehyde to GA is mainly ensured by a glycolaldehyde dehydrogenase encoded by *aldA* [64], the overexpression of which promoted increased GA production [63, 65]. However, not a single enzyme among the various aldehyde reductases that possesses *E. coli* [66] is devoted to the reduction of glycoaldehyde to EG. While Chae et al. [67] showed that the overexpression of *yqhD* encoding a broad-substrate NADPH-aldehyde reductase [68] was the most effective in the production yield of EG from xylose, Cabulong et al. [69] reported a better performance with *yjgB* also termed *ahr* encoding another broad-spectrum NADPH-aldehyde reductase. The discrepancy between these two reports is unclear. At variance to these studies, Wang et al. [70] showed that *fucO*, which encodes an NADH-aldehyde reductase, was the most efficient in the production of EG. These authors explained their results by considering a higher NADH availability in their engineered strain due to the deletion of *aldA* and *arcA,* a gene encoding a redox regulator under microaerobic condition and whose deletion results in derepression of oxygen-repressed genes [71]. Altogether, the NP_Dahms route to GA or EG comprises only five reaction steps from C5 sugars (Fig. 2), and only the last step of the pathway makes a distinction between the engineered *E. coli* strains for EG and for GA production.

A major problem observed upon implementation of this metabolic route in *E. coli* was a toxic accumulation of xylonic acid that strongly impairs growth. This also clearly identifies the xylonic dehydratase (XD) as rate-limiting enzyme of this pathway. Cabulong et al. [69] partially circumvented this problem by a combination of a weak expression of *xylB* encoding XDH together with an overexpression of *yjhG* encoding the endogenous XD. Reduction of *xylB* expression was also carried out by Chae et al. [67] using a synthetic small regulatory RNA inhibiting expression of this gene. Altogether, this five-step reaction of the NP_Dahms route to EG is redox neutral and can reach 50% of the yield efficiency (Y^E^) (Table 3). This Y^E^ of the pathway is calculated according to [72] as the ratio between the stoichiometric efficiency (or pathway yield) Y^p^ and the thermodynamic yield Y^th^, with the latter obtained as the ratio between the degree of reduction of the substrate to that of the final product. The fact that only 1 mol of EG is produced per mole of C5 sugar is due to the metabolic inability to recycle pyruvate into EG. This may also explain that this chemical cannot be produced from glucose at least using conventional pathways. Nonetheless, fermentation process has been established with *E. coli* equipped with the NP_Dahms pathway to reach titer of 108 g/L and productivity in the range of 2.25 g/L/h from d-xylose [67] (summarized in Additional file 1: Table S2).

**Table 3:** Yield and energy cost of bio-based chemicals according to production pathways expressed in *E. coli*

Bio-based products	Production pathway	Substrat	Nbr reaction step	NAD(P)H yield (mole/mole)	ATP yield (mole/mole)	Pathway yield (*Y*^p^)^$^	Yield efficiency (%)^*^
Ethylene glycol (EG)	NP-Dahms	Xylose	5	0	0	1	50
	Xylulose-1-P	Xylose	4	− 1	− 1	0.87	50
	Ribulose-1-P	Xylose	5	− 1	− 1	0.87	50
	Serine pathway	Glucose	13	+2	0	2	83.3
	X1P (R1P) + serine pathway	Xylose	7	+1	0	2	100
	X1P (R1P) + serine pathway	Xylose + glucose	11	+2	0	4	91
Glycolic acid (GA)	CCM- (GS)^$^	Glucose	18	+6	+2	2	66
	NP-Dahms	Xylose	5	+2	0	1	40
	Xylulose-1-P	Xylose	4	+1	− 1	0.96	40
	Ribulose-1-P	Xylose	5	+1	− 1	0.96	40
	NP-Dahms + GS	Xylose	10	+2 (+1QH2)	0	2	80
	X1P (R1P) + GS	Xylose	9	+ 3 + (1QH2	+1	2	80
	X1P (R1P) + GS	Xylose + glucose	15	+2 + (3 QH2)	+3	4	73
1,4 Butanediol (BDO)	CCM^$^	Glucose	21	0	0	1	92
	NP-Weimberg	Xylose	6	0	0	1	110
1,2,4 Butanetriol (BTO)	CCM^$^	Glucose	17	0	-1	1	83
	NP-KDC	Xylose	5	0	0	1	100
3,4 dihydroxybutyric acid (3,4-DHBA)	CCM	Glucose	22	+3	0	1	66
	NP-KDC	Xylose	5	+2	0	1	80
Mesaconic acid (MSA)	CCM^$^	Glucose	18	+3	+1	1	75
	NP-Weimberg	Xylose	8	+1	0	1	91
Glutaric acid (GTA)	CCM^$^	Glucose	17	+ 5.66	+1.33	1	
	NP-Weimberg + XI	Xylose	14	+4	0	0.66	62

XI means xylose isomerase pathway

^$^*Y*^p^ is the stoichiometry pathway efficiency strictly calculated from the pathway without balancing ATP and cofactors

*Yield efficiency is the ratio between the thermodynamic or energy yield and the pathway yield (*Y*^p^) as defined by [72]. The energy yield of these chemicals as produced from pentose or glucose can be found in Additional file 1: Table S4

^**$**^ CCM-GS means Carbon central metabolism—glyoxylate shunt

The yeast *S. cerevisiae* was also used as host to express the NP-Dahms pathway for EG production. To this end, the producer strain was constructed by genome integration of *C. crescentus xylB* under the strong *PGK* promoter while the other genes of the Dahms pathway, namely *xylD*, *yagE* and *aldA*, were expressed on a high-copy plasmid under the strong *TDH* promoter [73]. In addition, the strain was also deleted for competing pathways to avoid loss of xylose into xylitol. Upon incubation with d-xylose, the engineered strain only produced a tiny amount of EG, while exhibiting huge accumulation of d-xylonate as well as of 3-deoxypentanoic acid (3-DPA) and 3,4-dihydroxybutyric acid (3,4-3,4-DHBA). These two metabolites actually revealed the presence of endogenous aldo–keto reductase and 2-ketoacid decarboxylase that compete with KDPA for KDX, respectively (see below for further details). Their accumulation was mainly ascribed to the inefficient catalytic activity of xylonate dehydratase due to low availability of iron in yeast cytosol. Indeed, this enzyme belongs to LlvD/EDD protein family that requires iron for activity [74]. Support to this hypothesis was reported by Salusjarvi et al. [73] who found that the deletion of *FRA2* encoding a cytosolic protein implicated in the regulation of iron, resulted in a 13-fold increase of the XD activity, which was accompanied by a still modest increase of EG (14 mg/L) from d-xylose.

As already described above, GA is the other relevant bio-based product that can be obtained from pentose through the NP_Dahms. At first glance, it only requires the rerouting of glycolaldehyde by the overexpression of *aldA* encoding an NAD^+^-dependent glycolaldehyde reductase [64]. However, this pathway generates 2 mol of reduced cofactors (Table 3) that must be regenerated through the respiratory chain coupled with ATP synthesis. This energy can be spent for the pentose uptake and for cellular maintenance. Contrary to EG, GA can also be naturally produced in *E. coli* through the glyoxylate shunt (GS). Therefore, a linkage between GS and the NP_Dahms pathway at the level of pyruvate might be envisioned, resulting in the production of 2 mol GA per mole of pentose (Table 3). This linkage was attempted in two recent papers. In a first report [63], the connection of the two pathways was created from two different plasmids, termed module 1 and module 2, in an *E. coli* strain deleted for competing pathways, namely xylose isomerase (xylA), xylulose-5-P kinase (xylB) and glycolate oxidase (*glcDEF*). Module 1 was a low-copy plasmid carrying genes encoding the NP-Dahms pathway, i.e., *xdh*-*yagF*-*yagE*-*aldA* as well as *grhA* encoding a glyoxylate reductase (GLR). Module 2 was a high-copy plasmid carrying genes favoring GS, namely *aceA* encoding isocitrate lyase and *aceK,* which codes for the bifunctional isocitrate dehydrogenase kinase/phosphatase to reduce isocitrate dehydrogenase activity. A production of GA at 0.46 g/g d-xylose was obtained with this engineered *E. coli* strain. Same yield on d-xylose was obtained by Liu et al. [65] following a similar strategy except that *ackA* encoding acetate kinase was deleted instead of overexpressing *aceK*.

Glycolic acid can be also produced from glucose, by natural pathways that comprise no less than 13 steps, with a maximal stoichiometric yield of 2 (Table 3). Through conventional metabolic engineering, the carbon flux from glucose to glycolic acid can be favored, which merely implicates activation of the glyoxylate shunt and inactivation of competing pathways at the level of pyruvate and isocitrate. Hence, Deng et al. [75] engineered a GA producer *E. coli* strain from glucose that initially grew poorly on glucose, likely due to a high expression of *aceK* causing a blockage of TCA at the isocitrate dehydrogenase level. Following adaptive evolution by repeating transfer to mineral glucose (M9) medium, a producer strain with improved growth rate was isolated and shown to produce 54 g/L GA at 63% of the pathway yield (Additional file 1: Table S2). In a more recent report, the same authors reported a production of 65 g/L GA at a yield approaching 92% of the maximal pathway yield with an improved producer strain, which was obtained according to the following subtle genomic modifications. Expression levels of *aceA*, *ghrA* and *aceK* were optimized by placing these genes on a low-copy plasmid under the strong *trc* promoter. Flow of acetyl-COA into the initial TCA reaction was favored by overexpression of the citrate synthase encoding *gltA*. Competing pathway for pyruvate and GA consumption were deleted such as *ldhA* encoding the lactate dehydrogenase and *aldA* encoding the glycolaldehyde dehydrogenase. Finally, the fermentation process was optimized by adjusting the C/N ratio at 15:1 (summarized in Additional file 1: Table S2).

Production of GA from glucose by *Corynebacterium glutamicum* and yeasts has been also attempted [76, 77]. However, use of these microorganisms for this production presented several difficulties. For *C. glutamicum*, the maximal pathway yield (Y^P^) is 1.7 mol glycolic acid/mol glucose instead of 2 since not all NADPH is fulfilled by glycolysis. On the other hand, *C. glutamicum* does not possess a glyoxylate reductase. On top of that, glyoxylate shunt is inactive under glucose growth condition. Therefore, GA was produced using an engineered *C. glutamicum* deleted for *aceB* encoding malate synthase, attenuated for expression of *icd* encoding isocitrate dehydrogenase and overexpressing the *E. coli grhA* encoding glyoxylate reductase. Due to this attenuation of *icd*, which impairs TCA cycle, acetate had to be added in culture medium. A production of 5.3 g/L GA was obtained according to a 1:1 C-mole ratio between glucose and acetate [76]. The group of Pentillä et al. [77] reported the engineering of the glyoxylate shunt pathway in the yeast *S. cerevisiae* and *K. lactis* for GA production. The metabolic engineering in both yeasts requested to remove genes encoding malate synthase and cytosolic isocitrate dehydrogenase and to express the *A. thaliana GLYR1* encoding the glyoxylate reductase. d-Xylose was used as the carbon source to avoid glucose repression of the glyoxylate shunt [78]. The titer obtained by these engineered yeast was only 1 g/L with *S. cerevisiae* while it was 15 times higher with the engineered *K. lactis*. The observed difference was explained by the fact that *K. lactis* exhibits neither glucose repression of respiration nor a Crabtree effect, which is known to divert carbon and reducing power into ethanol [79].

### The NP_Weimberg metabolic route to yield BDO and other chemicals

In a second route, which we called NP_Weimberg pathway, the 2-keto-3-deoxy-(d/l)-pentanoate is dehydrated by a specific 2-keto-3-deoxy-d-xylonate dehydratase (KdxD) or a 2-keto-3-deoxy-l-arabinonate dehydratase (KdaD) to yield 2,5-dioxopentanoate (DOP). DOP is a strategic platform because it can be oxidized into α-ketoglutarate, from which a panel of other bio-based chemicals can be produced (Fig. 2). To find out an efficient NP_Weimberg pathway that could generate these chemicals, Zhang et al. [80, 81] devised an original screening strategy that employed an *E. coli* mutant defective in isocitrate dehydrogenase (∆*icd* mutant) unable to produce α-ketoglutarate from glucose by TCA. Therefore, growth rescue of this mutant on d-xylose or l-arabinose exclusively relied on the expression of genes clusters isolated from various Archea that harbor the NP_Weimberg pathway. An interesting result that came out from this screening was to find that the dehydratase enzyme encoded by *xylX* (for d-xylose degradation pathway) or *araE* (for l-arabinose degradation pathway) was clearly the bottleneck enzyme in this pathway.

As indicated in Table 3, mesaconic acid (MSA) can be produced from C5 sugars at a molar yield of 1 mol per mole of pentose. To achieve this production, Bai et al. [80] equipped *E. coli* strain with three compatible plasmids. Plasmid 1 (medium-copy) carried NP_Weimberg pathway genes to produce DOP from d-xylose, genes encoding glutamate mutase (GlmE) and methyl aspartate lyase (Mal) to generate mesaconate from DOP were cloned in a plasmid 2 (high-copy), and plasmid 3 (low-copy) carried four genes necessary for the regeneration and reactivation enzymatic system for coenzyme 12 that is essential for GlmE activity. This strain was moreover deleted for *yagE* and *yjhH* encoding KDPA to eliminate Dahms route and for *sucA* to avoid loss of α-ketoglutarate downstream in the TCA cycle. Altogether, the engineered strain was able to produce 14.7 g/L of MSA after 2 days at a yield of 0.74 g/g d-xylose, which corresponds to 85% of the pathway yield Y^P^ (Table 3). Although this pathway is ATP neutral, the extra NAD(P)H generated can be reoxidized through respiratory chain coupled with ATP synthesis to yield energy needed for sugar uptake and maintenance. Since the production of MSA by NP_Weimberg route is restricted to C5 sugars, Wang et al. [82] recently developed an MSA producer strain from glucose by rerouting carbon flux to α-KG via overexpression of *ppc, gltA* and *icd* encoding PEP carboxylase, citrate synthase and isocitrate dehydrogenase, respectively. In addition, PEP was made more available for PPC-dependent anaplerotic reactions by deletion of *pstG* and overexpression of *galP* and ppsA encoding sugar importer and PEP synthase. This engineered strain was able to produce 23.1 g/L MSA at a yield of 0.46 g/g glucose, which correspond to 64% of the pathway yield (Table 3 and Additional file 1: Table S2). The construction of an *E. coli* strain that harbors these two engineered pathways has not been reported yet, which would enable an MSA production from pentose and glucose at the same time.

Contrary to MSA, glutaric acid cannot be produced from pentose sugars by the NP-Weimberg pathway alone because this route cannot provide one of its key intermediates, i.e., acetyl-CoA. Indeed, de novo biosynthesis of glutaric acid from α-KG requires five steps (Fig. 2) and engages an acetyl-CoA at the first step catalyzed by homocitrate synthase. This first step as well as the two following ones have been implemented in *E. coli* from the leucine biosynthesis genes of the yeast *S. cerevisiae* [83], which enables α +1 carbon chain extension of α-ketoglutarate to yield α-adipate. This intermediate is further decarboxylated by a promiscuous α-keto acid decarboxylase encoded by *L. lactis kivD* to glutarate semi-aldehyde, and then oxidized into glutarate by a succinate semi-aldehyde dehydrogenase encoded by *gadB* from *P. putida* [84]. Since NP-Weimberg alone cannot produce glutaric acid, Wang et al. [85] conceived a strategy that assessed the synergistic effect of installing either one of combination of the three xylose pathways, namely xylose isomerase (XI), NP_Dahms and NP_Weimberg pathways in the production yield and titer of this organic acid. While the XI pathway has the potential to produce 0.56 mol glutaric acid per mol d-xylose, the NP_Weimberg pathway alone was unproductive because it cannot generate acetyl-CoA, whereas it can be provided by the NP_Dahms pathway via pyruvate (Fig. 2). However, the drawback of this branching pathway is an equimolar production of a C-2 compound glycolaldehyde that is converted into GA. Consequently, the maximal yield obtained by combining NP_Dahms and Weimberg is 0.5 mol of glutaric acid and 1 mol GA per mole of xylose. Hence, to increase the yield (thus to reduce loss of carbon as C0_2_), a combination of XI with NP_Weimberg should be the best since the calculated pathway yield of this arrangement is 0.625 mol glutaric acid per mole xylose. Wang et al. [85] tested this hypothesis by engineering *E. coli* with either one or a combination of two or of the three pathways. They found that the combination of NP_Weimberg and XI yielded the best producer strain, as expected from the model. Even though the titer was only 602 mg/L, it was 5 times and 2 times higher than using XI alone or NP_Weimberg + Dahms, respectively. Additional improvements were attempted by restricting XI pathway to the production of acetyl-CoA while NP_Weimberg was dedicated to production of α-ketoglutarate by deleting *gltA* encoding citrate synthase. Unexpectedly, these genetic interventions did not improve yield and titer, probably because they affected cell growth. In addition, a likely reason for low yield and titer of glutaric acid may be found in the promiscuity activity of the *a*-ketoacid decarboxylase encoded by *Lactococcus lactis kivD* as this enzyme can readily decarboxylate upstream intermediates of the pathway, namely 2-keto-3-deoxy-d-xylonate and DOP (see below). Thus, in addition to the problem of providing enough acetyl-CoA in the pathway [85], provision of α-ketoglutarate by the NP_Weimberg route could be another limitation that explains the low production yield of glutaric acid from d-xylose. Pathways engineering for de novo biosynthesis of glutaric acid from glucose via α-ketoglutarate by *E. coli* have been also reported [83]. A modest production of 0.3 g/L glutaric acid from 20 g/L glucose in 48 h was obtained, which corresponded to 10% of the pathway yield. Since this yield is 3.5-fold lower than that obtained from xylose using XI + NP_Weimberg, this argues that a potentially viable process for glutaric acid should request co-feeding strategy of C5 and C6 sugars combining CCM with NP-Weimberg pathways. Nevertheless, these engineered pathways are still far less efficient than the glutaric acid production build on l-lysine overproducing *C. glutamicum* strain. This lysine-based pathway offers a theoretical yield of 0.75 mol/mol glucose, which is higher than the α-ketoglutarate pathway (0.66 mol/mol glucose, Table 3). From lysine, only 4 additional enzymatic steps are needed which are a conversion of lysine to 5-aminovalerate by a lysine monoxygenase (DavB) and δ-aminovaleramidase (DavA), a transaminase to produce glutarate semialdehyde (GabT) and finally a glutarate semi-aldehyde dehydrogenase (gadB) to yield glutarate. While genes encoding these enzymes were overexpressed, high yield (75% of the maximal) and titer of glutaric acid (90 g/L, see Additional file 1: Table S2) in a fed-batch process were achieved thank to the identification of a 5-aminovalerate importer encoding gene whose overexpression resulted in a reimport of 5-aminovalerate [86].

The production of 1,4-butanediol (BDO) from C5 sugars has been also attempted through the NP-Weimberg pathway [81], and presents the advantage of requiring 3 times less enzymatic steps than that from glucose [87, 88]. From DOP, only two enzymatic steps catalyzed by α-keto acid decarboxylase and an aldehyde reductase, respectively, are requested (Fig. 2). Tai et al. [81] identified *Lactococcus lactis* KivD as the most efficient α-ketoacid decarboxylase (KDC) from an enzymatic screening of various bacterial 2-keto acid decarboxylases to convert DOP into butanedial. Assuming YqhD as the most active NADPH-dependent aldehyde reductase in *E. coli* [68], these authors reported the production of a few g/L of BDO from xylose in an *E. coli* strain equipped with the NP_Weimberg pathway genes, that also overexpressed *kivD* and was deleted for competing pathways (i.e., xylose isomerase, Dahms pathway). However, this engineered strain produced high amount of 1,2,4-butanetriol (BTO), whereas no BTO was produced using l-arabinose, as the carbon source. These results were explained by the fact that KDC from *Lactococcus lactis* was even more active on KDX than on DOP and was inactive on KDA [81]. Therefore, protein engineering of KivD was realized, leading to a KivD^V411I^ variant exhibiting catalytic efficiency 15-fold reduced on KDX and twofold increased on DOP [81]. Replacement of the wild-type KivD by KivD^V411I^ variant in the engineered *E. coli* equipped with the NP_Weimberg pathway resulted in a production of 0.37 g BDO per g d-xylose. Interestingly, this yield was in the same range as that obtained with a metabolically engineered *E. coli* strain for BDO production from glucose [88] (see Additional file 1: Table S2). This may indicate that there is plenty room for optimization of BDO production from pentose sugars by the NP_Weimberg pathway. However, this process is not expected to be economically viable due to the high cost of xylose used as the sole carbon source. On the other hand, the introduction of the NP_Weimberg pathway in an already good BDO producer strain from glucose [88] could increase the marketability of this organism for BDO production from lignocellulosic sugars.

### The NP_KDC-dependent metabolic route yielding BTO and 3,4-DHBA

The decarboxylation of the NP pathway intermediate KDX or KDA into (*R,S*) 3,4-dehydroxybutyraldehyde constitutes a third non-natural route that can lead to the production of 1,2,4-butanetriol (BTO) or 3,4-dihydrobutyric acid (3,4-DHBA). This metabolite can be further cyclized at acidic pH to give rise to 3-hydroxybutyrolactone (γHBL) (Fig. 3). These chemicals are widely exploited as precursors for polymers, plasticizer and pharmaceutical drugs (Table 2). The NP_KDC pathway has been initially established by Frost et al. [89]. These authors identified benzoylformate decarboxylase encoded by *mdlC* from *Pseudomonas putida* as able to decarboxylate KDX. They reported a production of 1.6 g/L BTO from 10 g/L xylonic acid by an *E. coli* DH5α strain carrying *mdlC* on a high-copy plasmid, while the two other reactions in the pathway were catalyzed by the endogenous xylonate dehydratase and aldehyde reductase. Subsequent works were dedicated to install an optimized NP_KDC pathway for pentose conversion into BTO or 3,4-DHBA in *E. coli*. This comprised the additional expression of a xylose (XDH) or arabinose dehydrogenase (ArDH) from *Caulobacter crescenti* (Table 1), selection of an appropriate aldehyde reductase or aldehyde dehydrogenase to produce BTO or 3,4-DHBA, or alternatively, expression of a CoA-acylating aldehyde dehydrogenase for the CoA-branch pathway of 3,4-DHBA (Fig. 3). This optimization also included the elimination of the Dahms route and d-xylose pathways into the pentose phosphate pathway [83, 90].

The expression of the NP_KDC pathway in *E. coli* led to the identification of bottlenecks at three enzymatic steps in the pathway and which may explain that titer and productivity of BTO and 3,4-DHBA are still far from any industrial exploitation (Additional file 1: Table S2). A first limitation was to find a huge accumulation and excretion of xylonic acid in *E. coli* equipped with the NP_KDC pathway. Only partial reduction of this co-product was achieved by overexpression of endogenous *E. coli yjhG* or *Caulobacter crescentus xylD* encoding xylonate dehydratase [90–94]. Since XD belongs to the IlvD/EDD protein family that requires iron–sulfur (Fe–S) cluster in its active center [74], availability of iron is essential to warrant efficient activity of XD, which likely becomes limiting when XD is highly overexpressed in the cell. A second issue dealt with the last step in the pathway to produce BTO or 3,4-DHBA, which engaged either an aldehyde reductase or an aldehyde dehydrogenase. This problem turned out to be very critical because all *E. coli* strains equipped with the NP_KDC pathway were found to produce both chemicals at once [83, 92, 95]. A study of Valdehuesa et al. [95] showed that the loss of function of *yqhD* almost completely abolished BTO production from d-xylose, but did not increase the other chemical. On the other hand, Wang et al. [93] screened six different *E. coli* aldehyde reductases and found that overexpression of an NADH-dependent aldehyde reductase encoded by *adhP* resulted in higher BTO titer. A possible explanation for these different results is that the two authors used different *E. coli* background strains to engineer the NP_KDC pathway. While Vadehuesa et al. [90] used an MG1655 derivative strain for which the overexpression of *yqhD* is detrimental for growth, Wang et al. [93] expressed the NP_KDC pathway in an *E. coli* BL21 strain that exhibits for unexplained reasons a higher capacity for BTO synthesis than MG1655. Nevertheless, the titer of BTO obtained with these various engineered *E. coli* expressing the NP-KDC pathway was in the range of 1 to 5 g/L with a yield that was at best 27% of the pathway yield (see Additional file 1: Table S2). To favor 3,4-DHBA at the expense of BTO, Wang et al. [96] overexpressed the endogenous *yneI* encoding a succinate semialdehyde dehydrogenase, which resulted in a twofold increase of 3,4-DHBA while levels of BTO were not reduced. An alternative strategy was to convert 3,4-dihydroxybutanal in its acyl-CoA derivative by a CoA-acylating aldehyde dehydrogenase followed by a thioesterase that releases CoA and 3,4-DHBA. This possibility was found to function in vivo but it did not improve titer and yield of the engineered strain [94] (see Additional file 1: Table S2). The high promiscuity and the low catalytic efficiency of the 2-keto-acid decarboxylase on KDX (and very likely on KDA) are the third issue that makes NP-KDC pathway still poorly effective for converting pentose into BTO and 3,4-DHBA. As indicated in Table 1, the catalytic efficiency of KDC on KDX is at least one order of magnitude lower than that of the other enzymes in the pathway. Until now, only few 2-keto-acid decarboxylases have been screened for their activity on KDX. According to Wang et al. [93], KdcA from *Lactococcus lactis* was found to be more active than KivD or MdlC in the production of BTO, whereas Gao et al. [94] found that production of 3,4-DHBA was equivalent whether the KDC was encoded by *L. lactis kivD* or *P. putida mdlC*.

The NP_KDC pathway has been recently expressed in the yeast *S. cerevisiae*. A production of 1.7 g/L of BTO from d-xylose at a yield of 25% (mol/mol) and 1.1 g/L from direct fermentation of rice straw hydrolysate have been reported [97]. The motivation to use yeast as a host organism for the production of this chemical resides in part in its capacity to ferment inexpensive complex hydrolysates obtained from mild acidic treatment of biomass, which are not tolerated by *E. coli* [98, 99]. A major issue in this work was to express an active xylonate dehydratase since this enzyme requires iron–sulfur (Fe–S) for activity [74], while cytosolic iron is rather low in yeast. Bamba et al. [97] showed that deletion of *BOL2* encoding a cytosolic protein involved in the repression of iron regulon [100] combined with the overexpression of *tTYW1*, whose effect was reported to increase cytosolic iron, resulted in an improved production of BTO from d-xylose with a 24% molar yield (see Additional file 1: Table S2).

Metabolic pathway has also been engineered in *E. coli* for the production of BTO and 3,4-DHBA or its lactone derivative from glucose [101–103]. As indicated in Fig. 3, the biosynthetic pathway of BTO from glucose comprises a natural part that corresponds to the metabolism of glucose into malate in 9 steps, and a non-natural route involving 6 reaction steps from malate to BTO. The non-natural pathway that converts malate to BTO was assembled using two compatible plasmids, each carrying a specific genes module. Genes of module 1 encode enzymes that convert malate into 2,4-dihydroxybutyric acid in 3 steps. The first step is catalyzed by a malate thiokinase (MTK) whose *mtkAB* gene was isolated from the archeabacterium *Methylibium petroleiphilum*. The produced malyl-CoA is then reduced into malate semialdehyde (MSA) by a succinate semialdehyde dehydrogenase encoded by *sucD* from *Porphyromonas gingivalis*. The last step is the reduction of MSA into 2,4-dihydroxybutyric acid that was found to be catalyzed by 4-hydroxybutyrate dehydrogenase encoded by *4*-*hbB* from *Porphyromonas gingivalis*. Module 2 was constructed with *abfT*-*2* of *Porphyromonos gingivalis* encoding 4-hydroxybutyrate COA transferase and *adhE2* of *Clostridium acetobutylicum* that encodes the bifunctional aldehyde/alcohol dehydrogenase. Upon transformation of *E. coli* strain BL21 with these two compatible plasmids, a tiny amount (120 ng/L) of BTO was detected. Although the feasibility of this non-natural pathway was demonstrated, many side products like BDO and 4-hydroxybutyrate were also accumulated, indicating that most of the requested enzymes for this non-natural pathway showed poor specificity for the intermediates of this pathway [101]. In addition, the requirement for reduced cofactors and ATP makes this pathway not beneficial with a maximal yield of 0.65 mol BTO/mol glucose (Table 3). Figure 3 also schematically illustrates the non-natural enzymatic steps leading to the synthesis of 3,4-DHBA from glucose. The whole pathway encompasses 15 enzymatic reactions, including a path of 5 non-natural reactions that converts glyoxylate to 3,4-DHBA. The construction of this pathway has been made using a transacetylase encoded by *pct* from *Megasphaera elsdenii*, a thiolase and acyl-CoA reductase encoded by *btkB* and *phaB*, respectively, from *Cupriavidus necator* and finally a thioesterase encoded by the endogenous *E. coli tesB* gene. In addition, overexpression of acetyl-CoA synthetase could be required to recover acetate into acetyl-CoA. Overall, this pathway could optimally produce 1 mol 3,4-DHBA from 1 mol glucose with the generation of 2 mol reduced equivalents and 1 mol ATP (Table 3). While this positive energetic balance is an advantage for strains’ stability and viability, major problem to be solved is many co-products generated due to the low specificity of the involved enzymes for their non-natural substrates. This is mainly the case for BtkB, which can condense two acetyl-CoA into 3-hydroxybutyryl CoA, or can produce equally 2 hydroxy-3-ketobutyryl-COA and 4-hydroxy-3-ketobutyryl-CoA from glycolyl-CoA and acetyl-coA, which thereafter generates equal amount of 2,3-3,4-DHBA and 3,4-3,4-DHBA [103]. Metabolic burden caused by the number of genes to assemble this pathway adds another difficulty that affects product titers. Altogether, the complexity of this glucose-depending pathway for the synthesis of 3,4-DHBA and γHBL raises question about its feasibility toward industrial development.

## Orthogonality of non-natural pentose-1-phosphate pathway to central carbon metabolism for optimal conversion of lignocellulosic sugars into bio-based products

The implementation of non-phosphorylating (NP) pathway in microbial systems for conversion of lignocellulosic sugars into bio-based products presents two main difficulties that may hinder use of these microbial strains for industrial exploitation. A first limitation is the strong dependency of the xylonate/arabinonate dehydratase to iron, which strongly affects in vivo activity, and explains high and eventually toxic accumulation of xylonic acid in engineered strains harboring the NP pathway. The second limitation relates to the fact that only pentose sugars can be converted into bio-based product through the NP. To circumvent the first limitation and to partially solve the second one, non-natural pathways have been recently designed that enable the production of bio-based products from lignocellulosic sugars at higher production yield and productivity. The example presented in this review will focus on the bioproduction of EG and GA.

**Fig. 4:**
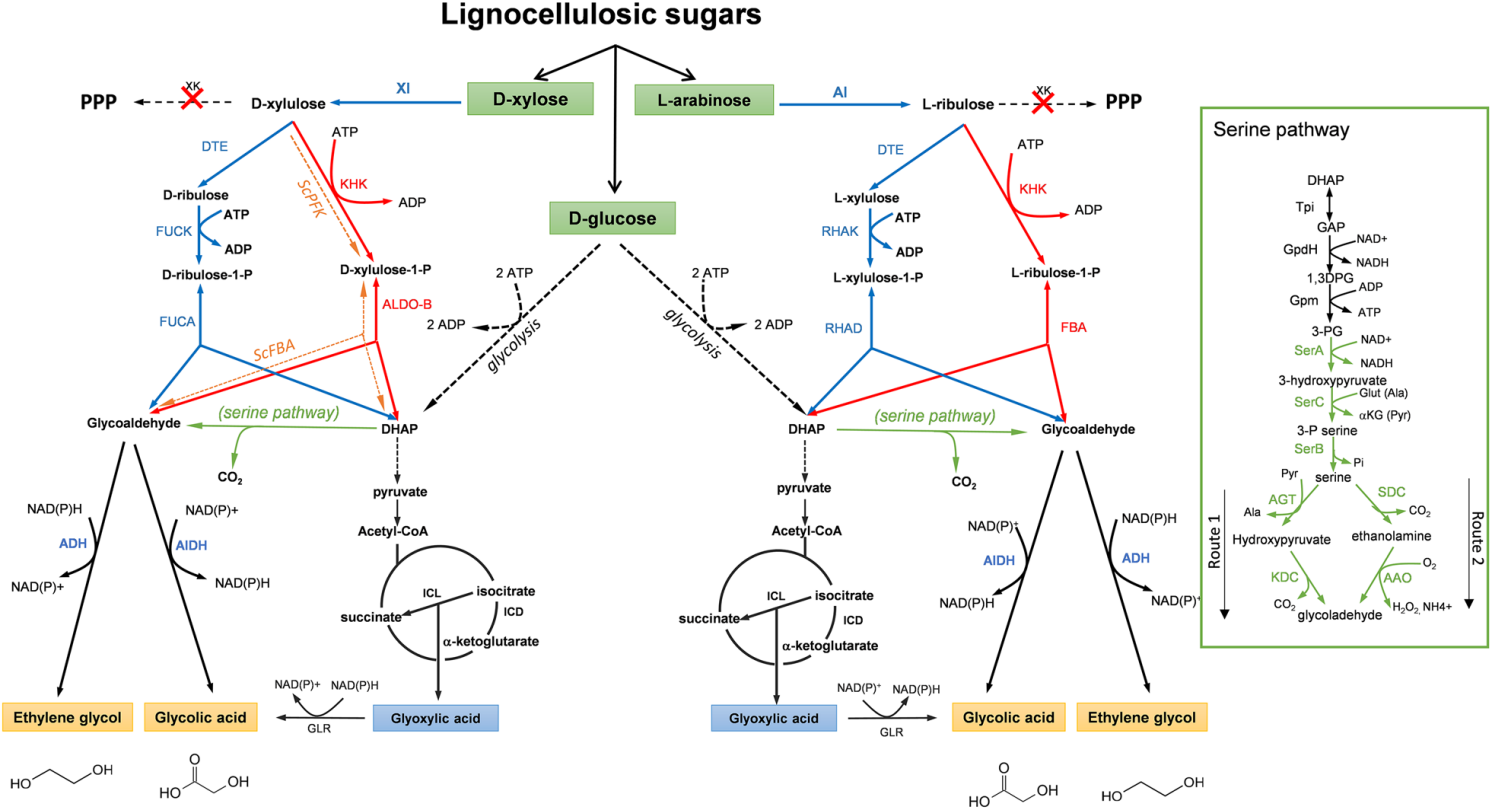
Xylulose 1-Phosphate, Ribulose-1-Phosphate and serine pathway connected to central carbon metabolism for conversion of pentose and hexose into GA and EG. XI: xylose isomerase; DTE: d-tagatose 3-epimerase; FUCK: fuculokinase: FUCA: fuculo-phosphate aldolase; KHKC: ketohexokinase C; Aldo-B: mammalian aldolase B; ScFBA: *S. cerevisiae* FBP aldolase; RHAK: L-rhamno-1-P kinase; RHAD: Rhamno-1-P aldolase; ICL: isocitrate lyase; AlDH: aldehyde dehydrogenase: ALR: aldehyde reductase. Inset of Fig. 4 is shown the two serine pathways. TPI: triose phosphate Isomerase; GpdH: glyceraldehyde-3-phosphate dehydrogenase; SerA: 3-hydroxypyruvate dehydrogenase; SerC: serine: α-KG transaminase; SerB: phosphoserine phosphatase; AGT: alanine-glyoxylate transaminase; KDC: α-keto-acid decarboxylase; SDC: serine αdecarboxylase; AAo: amino acid oxidase. Additional details on enzyme and encoding genes are given in Additional file 1: Table S3

As depicted in Fig. 4, three novel non-natural pathways have been designed, assembled and tested in vivo in *E. coli*, *C. glutamicum* or yeast for the production of EG and GA from pentose and glucose (also reviewed in [104]). The first two pathways draw their originality on the phosphorylation of the pentose sugar d-xylose or d-arabinose on its C-1 carbon instead of C-5 carbon as it occurs in the pentose phosphate pathway. Cam et al. [105] identified the mammalian ketohexokinase encoded by *khkC* [106] as very effective to phosphorylate d-xylose or l-arabinose into xylulose-1P or arabinose-1P, whereas Stephanopoulos’ group [107] trusted on the native fuculo-1-kinase and rhamnulokinase encoded by *fuck* and *araB* implicated in d-arabinose and l-lyxose metabolism [108] to yield similar pentose phosphorylation. However, the latter strategy required a step prior to phosphorylation that is catalyzed by a tagatose epimerase encoded by *dtE* from *Pseudomonas cichorii*, which converts d-xylulose into d-ribulose and l-ribulose into l-xylulose. The pentose-1P generated can be aldolytically cleaved into glycolaldehyde (C2) and DHAP (C3), which requires an aldolase that turns out to be rather specific for the pentose-1P produced. The mammalian aldolase B (AldoB) was found to be very efficient to cleave xylulose-1P, but ineffective on l-ribulose-1P [109], whereas endogenous aldolases encoded by *fucA* and *rhaD* were used to cleave d-ribulose-1P and l-xylulose-1P [107] (see Additional file 1: Table S3 for a summary of enzymes used in these non-natural pathways). Production of EG was found to be dependent on the aldehyde reductase encoded by *yqhD* but its maximal conversion from glycolaldehyde also needed overexpression of *fucO* and deletion of *aldA* [107, 110]. Conversely, maximal production of GA from glycolaldehyde [105] requires not only solely overexpression of *aldA* but also the deletion of *glcD* encoding the membrane-associated glycolate oxidase [111]. Expression of these non-natural pathways in *E. coli*, in which competing pathways of xylose and arabinose into PPP were deleted, resulted in the production of EG and GA that reached 85 to 90% of the maximal yield expected (1 mol/mol sugar) (see Additional file 1: Table S2). This 10 to 15% missing carbon raises the question of its fate from xylose since, at first glance, the issue of the two-carbon fraction of this sugar should be EG and/or GA.

Xylulose-1-Phosphate (X1P) and Ribulose-1-Phosphate (R1P) pathways have been tentatively expressed in the yeast *S. cerevisiae.* In a first report by [112], a weak production of 0.25 g/L EG was obtained with a ∆*xks1* yeast mutant defective in xylulose-5-kinase that overexpressed *XI*, *khkC* and *FBA1* encoding respectively xylose isomerase, mammalian ketohexokinase and the endogenous FBP aldolase. However, the production of EG was accompanied by a significant accumulation of xylitol, glycerol and ethanol. This finding indicated the presence of a very active aldo-reductase/alcohol dehydrogenase in spite of the fact that this engineered strain was deleted for *GRE3* encoding a major aldo-reductase implicated in the reduction of xylose into xylitol [113]. Another report revealed that *S. cerevisiae* may have a native capability to produce EG from glucose, given that it harbors a high xylose isomerase (XI) activity that has been obtained by in vivo evolutionary engineering. Because of this high XI activity, xylulose accumulates in the cells and can be illegitimately phosphorylated into its C1 carbon by phosphofructo-6-kinase encoded by *PFK1/2* [114]. In addition, the loss of function of the *XKS1*-encoded xylulose-5-P kinase favored GE production. A production of 4 g/L EG from 20 g/L xylose was obtained with such an XI-evolved ∆*xks1* strain that also overexpressed an *E. coli* aldehyde reductase encoded by *fucO* [114].

As stated above, a limitation of these non-natural pathways is that only the two-carbon fraction is diverted into the products, leaving the C3 fraction (DHAP) into the biomass. Thus, to increase the yield, it might be worth to recapture this C3 fraction to glycolaldehyde. A possibility is through the serine pathway, which can lead to glycolaldehyde from DHAP via 8 reaction steps from which the first five enzymatic reactions correspond to the natural pathway for serine biosynthesis. From serine, there are actually two different routes to yield glycolaldehyde (see inset Fig. 4). A first pathway (route 1) involves a deamination of serine into hydroxypyruvate by an aminotransferase (TA) or amino acid dehydrogenase followed by decarboxylation into glycolaldehyde by a α-ketoacid decarboxylase (KDC). This route will generate two extra NADH per DHAP produced. The second route (route 2) involves a decarboxylation of serine into ethanolamine by a serine decarboxylase (SDC) followed by a reduction into glycolaldehyde by a monoamineoxidase (AAO). This route will generate hydrogen peroxide in addition to NADH. In theory, the serine pathway shall yield 2 mol EG/mole glucose with 2 extra NADH (Table 3) that can be reoxidized through respiratory chain coupled to ATP synthesis. Pereira et al. [115] implemented the latter route into an *E. coli* strain and after some genetic modifications that was intended to favor the carbon flux in the serine pathway, the engineered strain produced 4.1 g/L EG from 20 g/L glucose which corresponded to 30% of the pathway yield (see Additional file 1: Table S2). Main reasons invoked for this relatively low yield were that serine is a major amino acid implicated in several essential metabolic pathways [116] and that C3 intermediate cannot be easily channeled into the serine pathway due to its central position in the carbon metabolism for growth [115]. In addition, this pathway generates H_2_O_2_ that can be toxic for the cell [117, 118]. As *Corynebacterium glutamicum* is a good producer of serine [119], Chen et al. [120] evaluated which one of the two serine pathways was the most efficient in the production of EG from glucose in this bacteria. This strategy required to identify an efficient α-ketoacid decarboxylase and a transaminase for route 1. The decarboxylase was screened by incubating a *C. glutamicum* strain that overexpressed the *E. coli* aldehyde reductase encoded by *yqhD* with hydroxypyruvate as the substrate to produce EG, whereas serine was used as the substrate for the transaminase. The benzoylformate decarboxylase encoded by *mdlC* from *P. putida* and alanine-glyoxylate transaminase from *Arabidopsis thaliana* (AtAGT) were retained by this screening test. Similarly, an active amino acid oxidase (AAO) and serine decarboxylase (SDC) were screened based on the production of EG using the same *C. glutamicum* strain as above but incubated with ethanolamine and serine, respectively. This screening identified the monoamineoxidase from *Arthrobacter* sp. and serine decarboxylase from A*rabidopsis thaliana* as the best candidates in route 2. Subsequently, these pathways were introduced in a *C. glutamicum* strain that had been engineered from hyperproduction of serine, notably by deletion of *sdA* encoding serine deaminase and overexpressing the *serABC* operon. These authors showed that a strain that expressed route 2 was roughly 3 times more efficient than that carrying route 1 for EG production from glucose. However, a higher production of EG was obtained in a strain that contained both route [120], even though the titer was still low (3.5 g/L) and the yield was only 12% of the maximal pathway yield (Additional file 1: Table S2), which is surprisingly lower than with the engineered *E. coli* constructed by Gu et al. [119].

While the X1P and R1P pathways were originally designed to convert C5 into bio-based products, the co-functioning of these synthetic pathways with the glyoxylate shunt (GS) has been investigated for the production of GA from both xylose and glucose. In theory, this combination shall produce 2 mol GA/mol of sugar with a positive redox and ATP balance (see Table 3). Preliminary attempts of GA production by the co-function of both pathways on d-xylose were awkward since Alkim et al. [109] did not find further increase of GA as compared to XIP pathway alone. On the contrary, Pereira et al. [107] reported a significant increased yield from 0.44 to 0.63 g/g xylose by combining R1P with GS pathway. A plausible explanation for this discrepancy is that the engineered strain made by Alkim et al. [109] was defective in *icd* encoding NADP^+^-dependent isocitrate dehydrogenase, the deletion of which is known to strongly impair TCA cycle [121]. This problem was overridden by a co-feeding of glucose and xylose which resulted in an improved GA yield up to 0.63 g/g total sugar with a yield on the xylose fraction that reached 75% of the maximal theoretical yield [109] (Table 3). Although an engineering strategy with serine pathway instead of GS has not been experimentally verified yet, one can anticipate that the combination of the X1P (or R1P) with serine pathway should also improve the EG production yield in co-feeding with xylose and glucose.

## Refactoring metabolic pathways for conversion of lignocellulosic sugars into bio-based products aimed at optimal carbon conservation

**Fig. 5:**
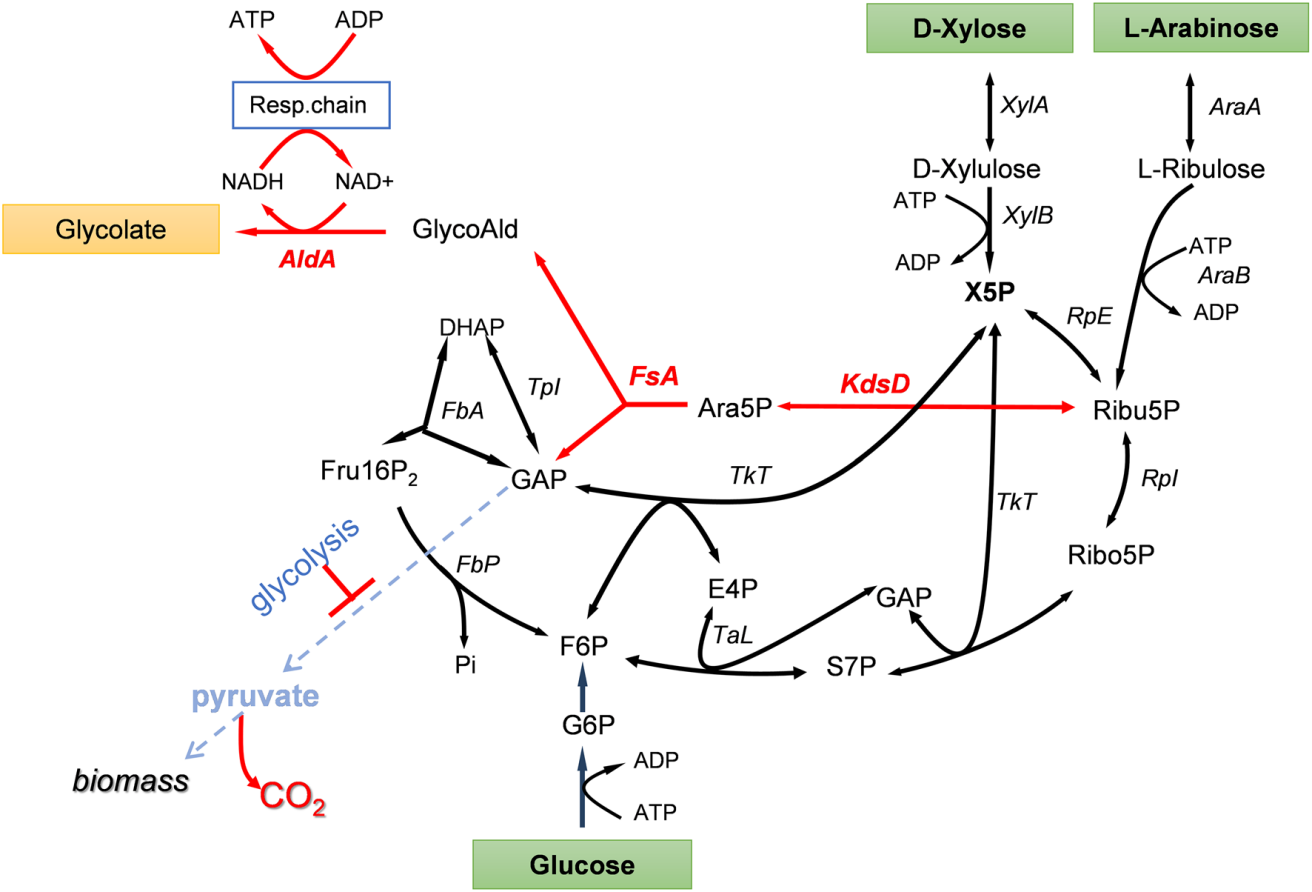
Scheme diagram of the glycoptimus pathway. Key steps arabinose-5-phosphate isomerase (KDSD) and Ara5P aldolase (FSA) are shown as red arrows. In green is shown the oxidation of glycoladehyde to glycolate by glycoladehyde dehydrogenase encoding by *aldA*. Other abbreviations are XylA: xylose isomerase; AraA: arabinose isomerase; XylB: xylulo 5-P kinase, AraB: arabinose-5-P kinase; RpE: ribulose 5-P-3 epimerase; RpI: Ribose isomerase; TkT: transketolase; TaL: transaldolase; TpI: triose isomerase; FbA: fructose-1,6P_2_ aldolase; FbP; fructose-1,6P_2_ phosphatase, KdsD: arabinose-5-P isomerase; FsA, fructose-6-PP aldolase; AldA: glycolaldehyde reductase

A general feature of metabolic pathways is their inherent carbon loss as CO_2_. There has been several attempts to refactor carbon metabolism in order to either overcome when possible or minimize this carbon lost and hence to optimize carbon conservation, as demonstrated by Liao and coworkers for the non-oxidative glycolysis (NOG). This reengineering of glycolytic pathway can lead to the maximal conversion 1 mol of glucose into 3 mol of acetate [122]. According to this objective, we recently designed a cyclic non-natural pathway—termed the glycoptimus pathway—enabling conversion of C5 and C6 sugar into GA with optimal carbon conservation (Fig. 5). The proposed pathway theoretically results in a yield of 3 mol GA per mole glucose or 2.5 mol GA per mole pentose, which is 50% higher than obtained by the natural-glyoxylate-dependent pathway. The key feature of glycoptimus relies on the use of arabinose-5P (Ara5P) isomerase encoded by *kdsD* and a class I aldolase encoded by *fsaA/fsaB* to generate glycolaldehyde and GAP from Xu5P. While glycolaldehyde is oxidized into GA, GAP can be shuttled back into the non-oxidative pentose phosphate pathway via transketolase and transaldolase reaction. This cycling scheme also generates 3 mol of NADH that needs to be reoxidized to allow a continuous running of the pathway. The reoxidation of NADH requires an active respiratory chain, which is coupled to the synthesis of ATP. This ATP provision is useful for the uptake and the phosphorylation of sugars, as well as for strain stability and maintenance. The in vitro and in vivo functions of the glycoptimus pathway have been validated although the yield of GA from glucose and xylose did not exceed 30% of the maximal yield expected. This poor efficiency of the pathway could be due to several problems and notably that the blockage of the lower part of glycolysis at the level of GAPDH may be detrimental for cell viability. In addition, many competitive pathways siphoning intermediates of the cycle have not been dismantled yet in the engineered strains and carbon flux in the *kdsD*-*fsaA* pathway needs to be optimised [123]. Finally, the reoxidation of NADH is likely to generate more ATP than needed for the sole functioning of the glycoptimus cycle, which could result in feedback inhibition due to accumulation of intermediates and eventually depletion of cofactors. Solving these issues together with the development of an appropriate fermentation process is critically important to develop an economically viable process of glycolic acid production from lignocellulosic sugars.

## Conclusion and prospective

The sustainability and competitiveness of biotechnological processes for the production of bio-based chemicals from renewable carbon sources critically depend on the need to equip the microbial platform with efficient metabolic pathways able to readily transform these carbon resources into bio-based products at titer, rate and yield (TRY) that are economically viable. For high-volume, low-priced chemicals such as those considered here, the higher these three performance indices are, the more competitive biotechnological processes become compared to petrochemical processes. Even though many microorganisms on earth are able to assimilate pentose and hexose sugars, their carbon metabolism is not optimally fashioned to achieve these performances. This necessitates to rewire their actual metabolic network, to plug (non-natural) pathways that exist in other organisms or to create new ones (artificial or synthetic), while ensuring cellular homoeostasis. Metabolic diversity found in Nature, combined with enzymes’ promiscuity, provides inspiration for implementing non-natural or constructing synthetic metabolic pathways that could meet these goals.

In this review, we showed that plugging the non-phosphorylating pentose pathway or implementing synthetic pentose-1-P pathway in the industrially tractable organism *E. coli*, *Corynebacterium glutamicum* or *S. cerevisiae* empower these microbial cells with strong capability to convert lignocellulosic sugars into a wide range of added-value chemicals. Although these microbial processes are far from industrial performances, there are strong indications that this objective could be soon reached. First, these pathways are orthogonal to the natural metabolic networks, which in theory make them insensitive to endogenous metabolic and genetic regulation, while being deliberately controllable by mean of biosensors/biocontrollers [124]. This orthogonality may allow to partition carbon utilization into the production and growth purpose, enabling tunable dynamic control to favor one purpose over the other, as it was demonstrated for the production of glucaric acid from glucose [125]. Second, the number of reaction steps from the substrate to the product in the constructed pathway has to be as short as possible. The synthetic pathway for BDO from xylose is a best example since it only encompasses 6 steps, instead of the 21 required from glucose. Less reaction steps go in hand with less optimization workload and less intermediates to be siphoned off in branching pathways. Consequently, higher yield and higher productivity should be expected for production of bio-based chemicals.

There still remain many challenges ahead to better improve microbial cell factories and concurrent biotechnology processes to make them industrially more appealing. We anticipate at least three major actions that must be actively worked on. The first one dealt with further rewiring of carbon metabolism for the conversion of all available renewable carbon resources, which include no solely lignocellulosic biomass, but CO_2_ and methane from landfills. Although the conversion of these carbon sources into bio-based chemicals still largely depends on traditional metabolic paths, new routes must be designed and constructed that should be orthogonal to the central carbon metabolism without perturbing the cellular homoeostasis. As for instance, new route for methane and CO_2_ assimilation into ethanol has been proposed by Liao and coworkers [126]. The same authors also provided various pathways enabling rewiring carbon metabolism to achieve maximal carbon conservation (reviewed in [127]). Innovation in yet inexistent pathways can be guided from exploration of the huge metagenomics data using chemoinformatics tools. This strategy has been recently applied to explore novel biosynthetic pathways for EG synthesis from C1 carbon source [128]. A second action that is critically determinant for high productivity and yield will be to increase catalytic efficiency and substrate specificity of pathway enzymes. However, getting these criteria can be a daunting task especially for enzymes in non-natural or synthetic pathways for which the substrates are not natural. This problem was clearly illustrated with α-ketoacid decarboxylase of *Lactococcus lactis*, whose promiscuity has been exploited to decarboxylate KdxD, DOP and α-adipate, but concomitantly generated metabolic side products due to this promiscuous activity [93]. Nevertheless, enzyme promiscuity provides an evolution starting point to yield specialized enzymes that catalyze the desired reactions on non-natural substrate. Rational enzyme engineering is a first approach to investigate the possibility to create enzyme with new function or with higher affinity to non-natural substrate than on its natural substrate. This strategy has been successful to completely change the specificity of an *E. coli* aspartate kinase encoded by *lysC* from its natural substrate—aspartate—to malate [129]. However, this rational approach is insufficient in many cases, asking for enzyme evolution either by random mutagenesis or by in vivo evolutionary engineering. Direct enzyme evolution can be very powerful if an appropriate high-throughput assay can be set up. Droplet-based microfluidic is thus well appropriate for this evolution, and its combination with IVTT (in vitro transcription–translation system) enables to screen for even larger libraries as it avoids transformation step in *E. coli* prior to the screen [130, 131]. Alternative to this method is the direct in vivo enzyme evolution using automated micro-reactors as developed by Altar company (http://www.altar.bio/about-us/). The use of this technique requires a phenotypic screening assay that exclusively relies on the vital function of the enzyme of interest. As an example, this strategy has been used to evolve an amino acid transaminase that can convert l-homoserine into the non-natural intermediate 2-keto-4-hydroxybutyrate (HOB), which thereafter could transfers its one carbon unit (CHO) to tetrahydrofolate (H4F), replacing the natural transfer C1-moieties from serine and glycine to H4F in an *E. coli* [132]. Fermentation process is likely a third target to be addressed early in the development of the microbial platforms as nicely reviewed by [133]. This aspect is really of the outmost importance when it deals with lignocellulosic hydrolysates or any raw materials that are on the one hand very cheap but on the other hand ill characterized, causing harsh fermentation conditions. Hence, key for industrial success, these new engineered microbial systems must be readily challenged with these harsh conditions, such as increasing their tolerance to toxic compounds and their global robustness by adapted laboratory evolution techniques [134, 135]. Besides these aspects, the choice between production phases coupled or decoupled to growth can be decisive in setting the fermentation process as it should be mostly determined by the capacity to reach maximal production yield, while assuring redox and energy balance.

## Supplementary information

**Additional file 1: Table S1.** Enzymes and corresponding genes that has been used in the expression of the that has been used in the Dahms, Weimberg and KDC-dependent non phosphorylating (NP) pathway for the production of bio-based commodity chemicals from hexose and pentose sugars by *E. coli* as described in Figs. 3 and 4. **Table S2.** Production titer and yield of bio-based chemicals from lignocellulosic sugars by engineered microorganisms. **Table S3.** Enzymes and corresponding genes that have been used in the xylulose-1P and ribulose-1-P synthetic pathways for the production of bio-based commodity chemicals from pentose sugars. **Table S4.** Thermodynamic yield and pathway yield of bio-based chemicals from sugars.

## Data Availability

Not applicable.

## Abbreviations

DHBA: 3,4-Dihydroxybutyric acid
3-DPA: 3-Deoxypentanoic acid
αKG: α-2-Ketoglutarate
AAO: Amino acid oxidase
Ara5P: Arabinose-5-phosphate
BDO: 1,4-Butanediol
BTO: 1,2,4-Butanetriol
CAGR: Compound annual growth rate
CCM: Central carbon metabolism
DHB: 2,4-Dihydroxybutyric acid
DOP: 2,5-Dioxopentanoate
EG: Ethylene glycol
GA: Glycolic acid
GAP: Glyceraldehylde-3-P
GLR: Glyoxylate reductase
H4F: Tetrahydrofolate
HOB: 2-Keto-4-Hydroxybutyrate
IVTT: In vitro transcription-translation system
kDa: 2-Keto-3-deoxy-l-arabinonate
KDC: α-Ketoacid decarboxylase (KDC)
KDP: d-2-Keto-3-deoxy-pentanoate
KDPA: 2-Keto-3-deoxy-pentanoate Aldolase
KDPH: 2-Keto-3-deoxy-pentanoate Hydratase
KDX: 2-Keto-3-deoxy-d-xylonate
KDA: 2-Keto-3-deoxy l-arabinonate
MSA: mesaconic acid
MTK: Malate thiokinase
NOG: Non-oxidative glycolysis
NP: Non phosphorylating pentose pathway
PEP: Phosphoenolpyruvate
PK: Phosphoketolase
PPP: Pentose phosphate pathway
R1P: Ribulose-1-Phosphate
SDC: Serine decarboxylase
TA: Transaminase
TCA: Tricarboxylic acid
TRY: Titer, rate and yield
X1P: Xylulose-1-Phosphate
XD: Xylonic dehydratase
XI: Xylose isomerase
Xu5P: Xylulose-5-Posphate
