# Supplementary material for: Engineering microbial pathways for production of bio-based chemicals from lignocellulosic sugars: current status and perspectives
Source: Biotechnol Biofuels. 2020 Jul 8;13:118. doi: 10.1186/s13068-020-01744-6 (PMC7341569; doi:10.1186/s13068-020-01744-6)
Supplement: Supplementary file 1 — Additional file 1: Table S1. Enzymes and corresponding genes that has been used in the expression of the that has been used in the Dahms, Weimberg and KDC-dependent non phosphorylating (NP) pathway for the production of bio-based commodity chemicals from hexose and pentose sugars by E. coli as described in Figs. 3 and 4. Table S2. Production titer and yield of bio-based chemicals from lignocellulosic sugars by engineered microorganisms. Table S3. Enzymes and corresponding genes that have been used in the xylulose-1P and ribulose-1-P synthetic pathways for the production of bio-based commodity chemicals from pentose sugars. Table S4. Thermodynamic yield and pathway yield of bio-based chemicals from sugars. [file 13068_2020_1744_MOESM1_ESM.docx]

Supplementary data

**Engineering microbial pathways for production of bio-based chemicals from lignocellulosic sugars:**

**Current status and perspectives**

Jean Marie FRANCOIS^1,2^, Ceren ALKIM^1,2^ and Nicolas Morin^1,2^

^1^Toulouse Biotechnology Institute, CNRS, INRA, INSA, Toulouse, France, 135 Avenue de Rangueil, F-31077 ^2^Toulouse, France; ^2^Toulouse White Biotechnology (TWB, UMS INRA / INSA / CNRS), NAPA CENTER Bât B - 3 rue Ariane 31520 Ramonville Saint-Agnes - France

**Key words:**

White biotechnology, Microbial physiology, metabolic engineering, synthetic biology, lignocellulose, glucose, xylose, arabinose, bio-based products, chemicals

Email address: [alkim@insa-toulouse.fr](mailto:alkim@insa-toulouse.fr)

[nicolas.morin@inra.fr](mailto:nicolas.morin@inra.fr)

[fran_jm@insa-toulouse.fr](mailto:fran_jm@insa-toulouse.fr) or [jean.marie-francois@inra.fr](mailto:jean.marie-francois@inra.fr)

*Correspondance to

Jean Marie François; TBI- INSA, 135 Avenue de Rangeuil, F-31077 Toulouse cedex 04 ;

Email: [fran_jm@insa-toulouse.fr](mailto:fran_jm@insa-toulouse.fr)

Phone: +33(0) 5 61 28 57 55

**Table S1 :** Enzymes and corresponding genes that has been used in the expression of the that has been used in the Dahms, Weimberg and KDC-dependent non phosphorylating (NP) pathway for the production of bio-based commodity chemicals from hexose and pentose sugars by *E.coli* as described in Figure 3 & 4.

| **Pathway** | **Enzyme name** | **Abbrev.** | **Gene name** | **Microbial origin** | **reference** |
| --- | --- | --- | --- | --- | --- |
| Common for NP-Dahms; NP-Weimberg, NP-KDC | Xylose dehydrogenase | XDH | xdh (xylB) | *Caulobacter crescentus* | [1 , 2-6] |
|  | Arabinose dehydrogenase | ArDH | araA  aadh | *Burkholderia multivorans*  *Pseudomonas fragis* | Tai, 2016 #6770}  [7] |
|  | Xylonolactonase | XL | xylC | *Caulobacter crescenti* | [4, 5, 8] [6] |
|  | arabonatelactonase | ArL | araB | *Burkholderia multivorans* | [6] |
|  | Xylonate dehydratase | XD | yagF/yjhG | *Escherichia coli* | [2-5, 8] |
|  | Arabonate dehydratase | ArD | araC | *Burkholderia multivorans* | [6] |
| NP-Dahms | 2-keto-3-deoxy-D-pentanoate aldolase | KDPA | yjhH  yagE | *Escherichia coli* | [1 , 2, 3 , 5, 9] |
|  | Glyoxylate reductase | GLR | ycdW/ghrA | *Escherichia coli* | [2, 3] |
| Common for  NP-Dahms and  NP-KDC | NAD^+^ - aldehyde dehydrogenase | AlDH | aldA | *Escherichia coli* | [2] |
|  | Aldehyde reductase -NADPH linked | ADR | yqhD  yjgB | *Escherichia coli*  *Escherichia coli* | [1 , 3 , 5] |
|  | Aldehyde reductase -NADH linked | ADR | fucO | *Escherichia coli* | [4] |
| NP-Weimberg | 2-keto-3-deoxy pentanoate dehydratase | KdxD  /KdaD | xylX_cc_  araD_bx_ | *Caulobacter crescentus*  *Burkholderia xenovorans* | [6] |
|  | 2-ketoglutarate semialdehyde dehydrogenase | KGSADH | xylA  araE | *C. crescentus*  *B. multivorans* | [6, 10, 11] |
|  | DOP decarboxylase | KDC | KivD  lpdC  mdlC | *Lactoccocus lactis*  *Salmonella typhimurium*  *Pseudomonas putida* | [6] |
|  | Glutamate dehydrogenase -NAD(P)+ dependent | GDH | gdhA | *Escherichia coli* |  |
|  | Glutamate mutase | GM | mutS/mutE  glmE/glmS | *Clostridium tetanomorphum*  *Clostrdium cochlearium* | [10, 12] |
|  | Methylaspartate ammonia lyase | MAL |  | *Clostridium tetanomorphum* | [12] |
|  | Homoaconitase | HA | *LYS4* | *Saccharomyces cerevisiae* | [11] |
|  | Homocitrate synthase | HCS | *LYS20* | *Saccharomyces cerevisiae* | [11] |
|  | Homoisocitrate dehydrogenase | HCDH | *LYS12* | *Saccharomyces cerevisiae* | [11] |
|  | Glutarate dehydrogenase (succinyl-semialdehyde dehydrogenase) | GlDB | gabB | *Escherichia coli* | [11] |
| NP-KDC | 2-Keto-acid decarboxylase | KDC | mdlC  kivD  kdcA | *Pseudomonas putida*  *Lactococcus lactis*  *Lactococcus lactis* | [7 , 13-15, 16 , 17 , 18] |
|  | Succinate semialdehyde dehydrogenase - NADP dependent | AlDH | yneI | *Escherichia coli* | [19] |
|  | CoenzymeA-acylating aldehyde dehydrogenase | CoA-AldH | pduP | *Lactococcus lactis*  *Klebsiella pneumonia* | [17] |
|  | Isocitrate lyase | ICL | aceA | *Escherichia coli* | [20] |
|  | Glyoxylate reductase | GLR | ghrA | *Escherichia coli* | [20] |
|  | Propionyl-CoA transferase | PCT | pct | *Megasphaera esdenii* | [20, 21] |
|  | thiolase | BtkB | *btkB* | *Cupriavidus necator* | [20, 21] |
|  | 3- hydroxybutyryl-CoA dehydrogenase | PhaB | *phaB* | *Cupriavidus necator* | [20, 21] |
|  | Acyl-CoA thioesterase | TesB | *tesB* | *Escherichia coli* | [20, 21] |
| CCM for BTO | malyl-CoA synthetase | MKT | mktAB | *Methylibium petroleiphum* | [22] |
|  | succinyl-COA reductase | SucD | sucD | *Porphyromona gingivalis* | [22] |
|  | 4 hydroxybutyrate dehydrogenase | 4hbD | 4hbd | *Porphyromona gingivalis* | [22] |
|  | 4-hydrobutyrate CoA transferase | Abft-2 | Abft-2 | *Porphyromona gingivalis* | [22] |
|  | bifunctional alaldehyde/ alcohol dehydrogenase | AdhE-2 | adhE-2 | *Clostridium acetobutylicum* | [22] |
| CCM for 3, 43,4-DHBA | Glyoxylate reductase | GLR | ghrA | *E. coli* | [20] |
|  | Propionyl-CoA transferase | PcT | pct | *Megasphaera elsdenii* | [20] |
|  | butyrylcoA thiolase | BktB | bktB | *Cupriavidus necator* | [20] |
|  | Butyryl-CoA reductase | BtR | phaB | *Cupriavidus necator* | [20] |
|  | Thioesterase II | TesB | tesB | *E.coli* | [20] |

**Table S2**: Production titer and yield of bio-based chemicals from lignocellulosic sugars by engineered microorganisms

| **Bio-based**  **chemicals** | **Host organism** | **genotypic characteristics** | **Experimental set-up** | **titer**  **(g/l)** | **% of max. yield** | **Productivity**  **(g/L/h)** | **Ref.** |
| --- | --- | --- | --- | --- | --- | --- | --- |
| Ethylene glycol | *E.coli* | *E.coli* W3110 engineered with the Dahms pathway (over expression of *xylB* from *C. crescenti* and *yqhD)* and deletion of *xylA* and *aldA (*strain EWXE4*)* | 5 L-batch fermentation, mineral medium M9 , 40 g/L xylose | 11.69 | 70* | 0.24 | [1] |
|  | *E.coli* | *E. coli* BL21 engineered with the Dahms pathway with overexpression of xylB and xylC from C. crescentus, overexpression of yjhH and fucO) and deletion of *arcA* and *aldA* (strain Q2843)) | 2-L Fed batch fermentation , mineral medium, feeding solution of 15 g/L xylose | 72 | 96* | 1.38 | [4] |
|  | *E.coli* | *E.coli* W3110 engineered with the Dahms pathway with overexpression of aldehyde reductase encoded by *yjgB* and regulated expression of xylose dehydrogenase encoded by xylB to avoid toxic accumulation of xylonic acid (strain WTXB) | 5 L-Batch fermentation, mineral medium, 20 g/l xylose | 7.72 | 95* | 0.06 | [3] |
|  | *E.coli* | *E.coli* W3110 engineered with the Dahms pathway (overexpression of xylB and xylC from C. crescentus and yqhD), deletion of xylA and modulation of xylB expression using anti-xylB sRNA to avoid toxic accumulation of D-xylonic acid (strain EG03 | 6,6-L Fed Batch fermentation with 2L- mineral medium, feeding solution of 700 G/l xylose | 108.2 | 87* | 2.25 | [5] |
|  | *E.coli* | *E.coli* MG1655 engineered with the Xylulose-1-P epimerase pathway. Expression of *pcdtE-fucK-fucA-fucO* and deletion of *aldA* and *xylB (*strain EG-4) | Fed batch fermentation, mineral medium M9, > 70 g/L xylose | 40 | 85* | 0.56 | [23] |
|  | *E.coli* | *E.coli* MG1655 engineered with the X1P kinase pathway, expression *khkC-aldoB-yqhD*; deletion of *xylB* and *aldA* (Strain Pen641) | Shake flask, mineral medium M9 with 10 g/L xylose | 3.88 | 94* | nd | [24] |
|  | *E.coli* | E.coli MG1655 engineered with the serine pathway (monoamine oxidase dependent): overexpression of *sdc* encoding *A. thaliana* Serine decarboxylase, *aao* encoding monoamineoxidase from Arthrobacter sp, *serB*, *serC* and *fucO* and deleted for *aldA* | Shake flasks, mineral medium M9 supplemented with 30g/L glucose | 4.1 | 20 | nd | [23] |
|  | *C. glutamicum* | Strain PABS1 engineered with the two serine pathways *ie* expression of AtAGT-mdlC-yqhD on a pEC-P1 plasmid and Atsdc-ASAO-yqhD on plasmid pCRB-P1 and deleted for sdA encoding serine deaminase | 5-L batch fermenter, with 2L-working volume containing mineral medium and supplemented with 40 g/L glucose | 3.5 | 12.5 | nd | [25] |
|  | *Yeast* | *S. cerevisiae* D452-2 strain engineered with X1P pathway; ie expression of mammalian *khkC*, overexpression of endogenous *FBA1* and deletion of *XKS1* | Shake flask containing minimal medium pH 6.0 supplemented with 40 g/l xylose | 0.2 | < 2 | nd | [26] |
| Glycolic acid | *Yeast* | *S. cerevisiae CENPK 113-7A* engineered with the NP-Dahms pathway ie expressing xylB, xylD from C. crescenti, *yagE and aldA* from E.coli and further deleted for *FRA2* | Shake flask in synthetic complete medium (SD) containing 10g/L glucose and 20g/L xylose | 1 |  | nd | [8] |
|  | *E.coli* | *E.coli* W3110 engineered with Dahms pathway linked to glyoxylate shunt: Overexpression of xdh/*xylB* from *C. crescenti*, *yagE* and *yagF* and *ycdW/grhA* on a module 1 (low copy plasmid), *aceA* and *aceK* as module 2 on a high copy plasmid, deletion of *xylAB* and *glcDEF*. | Shake flaks, mineral medium M9 + 1g/L peptone, 0,5 g/L yeast extract and 10 g/L xylose | 4.57 | 46 | nd | [2] |
|  | *E.coli* | *E.coli BL21* engineered with the Dahms pathway linked to glyoxylate shunt: overexpression of *xdH-xylC-yjhH* on plasmid 1 and *aldA-aceA-yjhG* on plasmid 2, with deletion of ackA |  | 43.6 | 46 | 0.91 | [9] |
|  | *E.coli* | *E. coli* BW25113 in which *aceA*, aceK and *grhA* have been integrated at the *glcB* locus under PLac promoter, also deleted for *aceB* and *glcB* and which has been subjected to adaptive evolution | 5-L bioreactor Fed-batch fermentation, in M9 medium feeding solution was 130 g/L glucose | 56.4 | 63 | nd | [27] |
|  | *E.coli* | *E.coli* Mgly434 which is deleted for aceB, glcB, aldA and ldhA and bears on a pBR322 plasmid aceA-aceK-ghrA | 5-L bioreactor, fed batch fermentation using a feeding solution of concentrated glucose and ammonium chloride at a ratio of 15:1 | 65.5 | 92 | nd | [28] |
|  | *E.coli* | *E. coli* MG1655 engineered with the synthetic X1P kinase pathway, expression of *khkC-aldoB-aldA*; deletion of *xylB*, and *glcD (*strain | Shake flasks, mineral medium M9 + 10 g/L xylose | 4.30 | 92* | nd | [29] |
|  | *E.coli* | *E.coli* MG1655 engineered with the synthetic xylulose-1-P epimerase (dte) pathway, expression of *pc.dtE-fucK-fucA-aldA*; deletion xylB and *glcD* | Batch fermentation, mineral medium M9 with > 100g/L xylose | 44 | 92* | nd | [23] |
|  | *E.coli* | *E.coli* MG1655 engineered with the synthetic xylulose-1-P epimerase (*dtE*) pathway and the glyoxylate shunt. expression of *pcdtE-fucK-fucA-aldA*; deletion *xylB* and *glcD*; overexpression of ghrA-aceA-aceK and deletion of aceB/glcB, gcl and xylB | Batch fermentation, mineral medium M9 with 65 g/L xylose and glucose | 40 | 63 | nd | [23] |
|  | *E.coli* | *E coli* MG1655 engineered with XIP pathway and glyoxylate shunt. Expression of *khkC-aldoB-aldA*, overexpression of *ghrA* and *aceA*; deletion of *xylB*, *aceB, gcL, glcD, iclR, edd-eda, acrA, icd* | Shake flasks, mineral medium M9 + 2 g/L tryptone, 2 g/L glucose and 5 g/L xylose | 3.73 | 75* | nd | [30] |
|  | *C. glutamicum* | *C. glutamicum ATCC13032* engineered with GS pathway: deletion aceB, attenuation icd(A1T), overexpression E;coli ycdW | Shake flasks, CGXII mineral medium at 30°C with 25 mM glucose/75 mM acetate | 5.3 | na | nd | [31] |
| 1, 4 Butanediol | *E. coli* | *E.coli* BW25113 engineered withNP-Weimberg pathway (ECKh-422). Expression of the xylBCDX operon of C. crescentus, deletion of *E. coli* *yjhH,* *yagE* and xylA and overexpression of kivD^V461I^ variant of *L. lactis* and *E. coli* YqhD | batch fermentation, mineral medium M9, 10 g/L D-xylose | 3.88 | 61 | nd | [10] |

|  | *E.coli* | *E.coli* BW25113engineered with NP-Weimberg pathway. Expression of the xylBCDX operon of *C. crescenti* as well as overexpression of xylA of C. crescentus; deletion of yjhH,yagE and araA and overexpression of kivD^V461I^ variant of *L. lactis* and YqhD | Fed-batch fermentation, mineral medium M9, 70 g/L L-arabinose | 15.6 | 37 | nd | [10] |
| --- | --- | --- | --- | --- | --- | --- | --- |
|  | *Yeast* | *S. cerevisiae YPH499* engineered with the NP-KDC pathway: overexpression of C. Crescentus xylB and xylD, of *Lactococcus lactic* *kdcA*, and yeast *yTYW1* and deleted for *GRE3* and *BOL2* | Shake flash, 30°C, rich medium –YP) with 10 g/L glucose and 10 g/L xylose | 1.7 | 24% (on D-xylose) | nd | [32] |
| 1,2,4 Butanetriol | *E.coli* | *E.coli* W3110 engineered with NP-KDC pathway. Overexpression of *xylB* from *C. crescenti*, of *mdlC* from *P. putida* and adhP from *E.coli*. Deletion of *xylAB*, *yjhH* and *yagE* | Shake flask fermentation. Mineral medium with 10 g/L D-xylose | 0.88  *(+ 8,8 g/l xylonic acid*) | 12 | nd | [13] |
|  | *E.coli* | *E.coli* BL21 engineered with NP-KDC. Overexpression of *xylB*, *xylC* from *C. crescenti*, of *mdlC* from P. putida *yjhG* and adhP from *Ecoli*. Deletion of *xylAB, yijH and yagE* | 5-L batch fermentataion, Mineral medium 20 g/L D-xylose + | 3.92 | 27.7 | nd | [15] |
|  | *E.coli* | *E.coli* BW2511 engineered with the NP-KDC pathway Overexpression of *xdh* from *C. crescenti*, *mdlC* from *P. putida* and *adhP* of *E coli*, deletion of *xylA, xylB, yjhH, yagE, yiaE* and *ycdW* | 500 ml Shake flasks fermentation  Mineral medium M9 with 20 g/l D-xylose | 1.58  *(+13.8 g/l xylonic acid*) | 7.9 | nd | [16] |
|  | *E.coli* | *E coli* BL21 (DE3) engineered with NP-KDC pathway (= strain BL21-14) . Overexpression of xylB(xdH), yjhG, mldC, xylD and adhP. This strain is also defective in the Dahms pathway | 500-ml shake-flask fermentation in LB medium with 40g/L xylose and antibiotic. Induction of the pathway by IPTG | 5.1 | 12.7 | 0.13 | [18] |
|  | *Yeast* | Strain BDδD-2tkcdA from YP499 that carries Caulobacter crescentis *xylD* and *xylB* integrated in the genome xylD was integrated at multi copy using δ-sequence), also 2 copies of K lactis *kcdA* encoding 2-keto acid decarboxylase, deleted for *GRE3* (a xylose dehydrogenase), and overexpression t*TYW1 encoding* the truncated form of Tyw1P which allows higher cytosolic iron | Batch fermentation in a YP rich medium supplemented with 10 g/L glucose and 10 g/L xylose | 1.7 | 24 | nd | [32] |
| 3,4 dihydroxybutyric acid  /3 hydroxybutyrolactone | *E.coli* | *E. coli* BW2511 engineered with the NP _KDC pathway. Expression of *xylBCD* of *C. crescenti,* *kivD* of *L. lactis*, *yneI* of *E coli* and deletion of *xylAB*, *yjhH* and *yagE* | Shake flask fermentation  LB modified with 20 g/l D- xylose | 1.27 | ND | nd | [19] |
|  | *E.coli* | *E.coli* MJ33-K1 engineered with the NP-KDC pathway. Expression of *xylB* and *xylD* of C. crescenti, *mdlC* of *P putida*. Overexpression of *feaB*, *ycdW* and *noxE*. Deletion of *yqhD*, *fucO*, *adhE*, *adhP*, *mgsA*, *yiaE*, *yagE* and *yjhH* *of E.coli* | Shake flask fermentation  LB medium with 20 g/L D-xylose and 5 g/l glucose | 0.39 | ND | nd | [17] |
| Glutaric acid | *E.coli* | *E.coli* BW25113 engineered with combination of Xylose isomerase and NP-Weimberg pathway. Expression of *xylBCDX* of *C. crescentus*, deletion of *yagE* and *yghH*. Overexpression of HCS, HA, HiCDH of *S. cerevisia*e, *kivD* from *L. lactis* and *gabD* from *P. putida* | Shake flask fermentation  LB medium  10 g/L D-xylose | 0.6 | 10 | nd | [11] |
|  | *C. glutamicum* | Strain GTA-4 derivated from a metabolically engineered producer of 5-aminovalerate , which gabTD operon encoding encoding aminovalerate transaminase and glutarate semialdehyde dehydrogenase under the strong *tuf* promoter and bears two copies in the genome of NCgl0464 under tuf promoter encoding 5-aminovalerate importer | 1L -Fed Batch fermentation, glucose-molasses (initially 50 g/L glucose, 72 .4 g/l sugar cane molasses, and then feed with a solution of 500g/L glucose; 162,5 g/l sugar cane molasses, in mineral medium | 90 | 75 | 1.8 | [33] |
| Mesaconic acid | *E.coli* | *E.coli* BW25113 engineered with NP-Weimberg pathway. Expression of *xylBCDX* operon of *B. xenovorans*. Overexpression of *glmE* and MAL encoding glutamate mutase and methylaspartate ammonia lyase. Overexpression of *araE* encoding a pentose /proton symporter | Shake flask fermentation. Mineral medium buffered with CaC0_3_  20 g/L D-xylose | 14.7 | 87 | nd | [10] |
|  | *E.coli* | *E.coli* BW25113 engineered for mesaconate production from glucose by expressing GM, MAL and reactivatase of GM (all genes carried on two compatible plasmids) | Shake flask fermentation in M9 medium complemented with 5 µM adenosylcobalamin and in the presence of 35 g/L | 6.9 | 27 | 0.25 | [12] |
|  | *E.coli* | *E.coli BW25113* engineered for mesaconate production from glucose by overexpression of GM, MAL and reactivatase of GM (for mesaconate pathway), overexpressing also *galP-gltA-icd-acnA-ppsA* and further deleted of *sucA, ptsG fumA pckA* | Shake flask fermentation, pH 7.2 buffered with CaC03, M9 medium supplemented with 5g/L yeast extract, 5µM coenzyme B12, 50 g/L glucose | 23 | 64 | nd | [34] |

**maximal yield is based on the pathway stoichiometry*

**Table S3**: Enzymes and corresponding genes that has been used in the xylulose-1P and ribulose-1-P synthetic pathways for the production of bio-based commodity chemicals from pentose sugars

| Enzyme name | Abbreviation | Gene name | Microbial origin | reference |
| --- | --- | --- | --- | --- |
| Xylose isomerase | XI | xylA | *Escherichia coli* | [30, 35, 36] |
| Arabinose isomerase | AI | araA | *Escherichia coli* | [35] |
| D-tagatose -3-epimerase | DTE | dtE | *Pseudomonas cichorii* | [23] |
| L-fuculokinase | FUCK | fucK | *Escherichia coli* | [23] |
| L-rhamnulokinase | RHAK | rhaB | *Escherichia coli* | [23] |
| L-rhamnulo-1-Phosphate aldolase | RHAD | rhaD | *Escherichia coli* | [23] |
| L-fuculose-phosphate aldolase | FUCA | fucA | *Escherichia coli* | [23] |
| Ketohexokinase C | KHKC | khkC | *Homo sapiens* | [30, 36] |
| Adolase type B | ALDoB | aldoB | *Homo sapiens* | [30, 36] |
| Aldehyde reductase -  NADPH linked | ADR | yqhD  yjgB | *Escherichia coli* | [24] |
| Aldehyde reductase  -NADH linked | ADR | fucO | *Escherichia coli* | [35] |
| Glyoxylate reductase | GLR | ycdW/ghrA | *Escherichia coli* | [35]  [30] |
| Aldehyde dehydrogenase A NAD^+^linked | ALD | aldA | *Escherichia coli* | [35]  [29] |
| Isocitrate lyase | ICL | aceA | *Escherichia coli* | [30] |

**Table S4**: Thermodynamic yield and pathway yield of bio-based chemicals from sugars

| **products** | **MW**  **g/mole** | **Chemical formula** | **Degree of reduction** | **Thermodynamic yield Y^th^** | |
| --- | --- | --- | --- | --- | --- |
|  |  |  |  | glucose | pentose |
| xylose | 150 | C_5_H_10_O_5_ | 20 | _ | _ |
| glucose | 180 | C_6_H_12_O_6_ | 24 | - | - |
| ethylene glycol | 62 | C_2_H_6_O_2_ | 10 | 2.4 | 2.0 |
| glycolic acid | 76 | C_2_H_4_O_3_ | 6 | 4 | 3.33 |
| glyoxylic acid | 74 | C_2_H_2_O_3_ | 4 | 6 | 5 |
| 1,4 butanediol | 90 | C_4_H_10_O_2_ | 22 | 1.09 | 0.9 |
| 3,4 dihydroxybutyric acid | 120 | C_4_H_8_O_4_ | 16 | 1.5 | 1.25 |
| 2,4 dihydroxybutyric acid | 120 | C_4_H_8_O_4_ | 16 | 1.5 | 1.25 |
| glutaric acid | 132 | C_5_H_8_O_4_ | 20 | 1.2 | 1.0 |
| mesaconic acid | 130 | C_4_H_6_O_4_ | 15 | 1.33 | 1.11 |
| 1,2,4-butanetriol | 106 | C_4_H_10_O_3_ | 20 | 1.2 | 1.0 |
| ethanol | 46 | C_2_H_6_O | 12 | 2 | 1.67 |
| Acetic acid | 60 | C_2_H_4_O_2_ | 8 | 3 | 2.5 |

Thermodynamic yield Y^th^ = ratio between degree reduction of sugar/ degree of reduction of the product

**References**

1. Liu H, Ramos KR, Valdehuesa KN, Nisola GM, Lee WK, Chung WJ: **Biosynthesis of ethylene glycol in Escherichia coli.** *Appl Microbiol Biotechnol* 2013, **97:**3409-3417.

2. Cabulong RB, Lee WK, Banares AB, Ramos KRM, Nisola GM, Valdehuesa KNG, Chung WJ: **Engineering Escherichia coli for glycolic acid production from D-xylose through the Dahms pathway and glyoxylate bypass.** *Appl Microbiol Biotechnol* 2018, **102:**2179-2189.

3. Cabulong RB, Valdehuesa KN, Ramos KR, Nisola GM, Lee WK, Lee CR, Chung WJ: **Enhanced yield of ethylene glycol production from d-xylose by pathway optimization in Escherichia coli.** *Enzyme Microb Technol* 2017, **97:**11-20.

4. Wang Y, Xian M, Feng X, Liu M, Zhao G: **Biosynthesis of ethylene glycol from d-xylose in recombinant Escherichia coli.** *Bioengineered* 2018, **9:**233-241.

5. Chae TU, Choi SY, Ryu JY, Lee SY: **Production of ethylene glycol from xylose by metabolically engineered Escherichia coli.** *AIChE Journal* 2018, **64:**4193-4200.

6. Tai YS, Xiong M, Jambunathan P, Wang J, Wang J, Stapleton C, Zhang K: **Engineering nonphosphorylative metabolism to generate lignocellulose-derived products.** *Nat Chem Biol* 2016, **12:**247-253.

7. Niu W, Molefe MN, Frost JW: **Microbial synthesis of the energetic material precursor 1,2,4-butanetriol.** *J Am Chem Soc* 2003, **125:**12998-12999.

8. Salusjarvi L, Toivari M, Vehkomaki ML, Koivistoinen O, Mojzita D, Niemela K, Penttila M, Ruohonen L: **Production of ethylene glycol or glycolic acid from D-xylose in Saccharomyces cerevisiae.** *Appl Microbiol Biotechnol* 2017, **101:**8151-8163.

9. Liu M, Ding Y, Xian M, Zhao G: **Metabolic engineering of a xylose pathway for biotechnological production of glycolate in Escherichia coli.** *Microb Cell Fact* 2018, **17:**51.

10. Bai W, Tai YS, Wang J, Wang J, Jambunathan P, Fox KJ, Zhang K: **Engineering nonphosphorylative metabolism to synthesize mesaconate from lignocellulosic sugars in Escherichia coli.** *Metab Eng* 2016, **38:**285-292.

11. Wang J, Shen X, Lin Y, Chen Z, Yang Y, Yuan Q, Yan Y: **Investigation of the Synergetic Effect of Xylose Metabolic Pathways on the Production of Glutaric Acid.** *ACS Synth Biol* 2018, **7:**24-29.

12. Wang J, Zhang K: **Production of mesaconate in Escherichia coli by engineered glutamate mutase pathway.** *Metab Eng* 2015, **30:**190-196.

13. Valdehuesa KNL, H., Ramos, K.R.M., Park, S.J., Nisola, G/M., Lee, W-K., Chung, W-J: **Direct bioconversion of D-xylose to 1,2,4-butanetriol in an engineered Escherichia coli.** *Process Biochem* 2014, **49:**25.

14. Valdehuesa KN, Lee WK, Ramos KR, Cabulong RB, Choi J, Liu H, Nisola GM, Chung WJ: **Identification of aldehyde reductase catalyzing the terminal step for conversion of xylose to butanetriol in engineered Escherichia coli.** *Bioprocess Biosyst Eng* 2015, **38:**1761-1772.

15. Cao Y, Niu W, Guo J, Xian M, Liu H: **Biotechnological production of 1,2,4-butanetriol: An efficient process to synthesize energetic material precursor from renewable biomass.** *Sci Rep* 2015, **5:**18149.

16. Sun L, Yang F, Sun H, Zhu T, Li X, Li Y, Xu Z, Zhang Y: **Synthetic pathway optimization for improved 1,2,4-butanetriol production.** *J Ind Microbiol Biotechnol* 2016, **43:**67-78.

17. Gao H, Gao Y, Dong R: **Enhanced biosynthesis of 3,4-dihydroxybutyric acid by engineered Escherichia coli in a dual-substrate system.** *Bioresour Technol* 2017, **245:**794-800.

18. Wang X, Xu N, Hu S, Yang J, Gao Q, Xu S, Chen K, Ouyang P: **d-1,2,4-Butanetriol production from renewable biomass with optimization of synthetic pathway in engineered Escherichia coli.** *Bioresour Technol* 2018, **250:**406-412.

19. Wang J, Shen X, Jain R, Wang J, Yuan Q, Yan Y: **Establishing a novel biosynthetic pathway for the production of 3,4-dihydroxybutyric acid from xylose in Escherichia coli.** *Metab Eng* 2017, **41:**39-45.

20. Dhamankar H, Tarasova Y, Martin CH, Prather KL: **Engineering E. coli for the biosynthesis of 3-hydroxy-gamma-butyrolactone (3HBL) and 3,4-dihydroxybutyric acid (3,4-DHBA) as value-added chemicals from glucose as a sole carbon source.** *Metab Eng* 2014, **25:**72-81.

21. Martin CH, Dhamankar H, Tseng HC, Sheppard MJ, Reisch CR, Prather KL: **A platform pathway for production of 3-hydroxyacids provides a biosynthetic route to 3-hydroxy-gamma-butyrolactone.** *Nat Commun* 2013, **4:**1414.

22. Li X, Cai Z, Li Y, Zhang Y: **Design and construction of a non-natural malate to 1,2,4-butanetriol pathway creates possibility to produce 1,2,4-butanetriol from glucose.** *Sci Rep* 2014, **4:**5541.

23. Pereira B, Zhang H, De MM, Lim CG, Li ZJ, Stephanopoulos G: **Engineering a novel biosynthetic pathway in Escherichia coli for production of renewable ethylene glycol.** *Biotechnol Bioeng* 2016, **113:**376-383.

24. Alkim C, Cam Y, Trichez D, Auriol C, Spina L, Vax A, Bartolo F, Besse P, Francois JM, Walther T: **Optimization of ethylene glycol production from (D)-xylose via a synthetic pathway implemented in Escherichia coli.** *Microb Cell Fact* 2015, **14:**127.

25. Chen Z, Huang J, Wu Y, Liu D: **Metabolic engineering of Corynebacterium glutamicum for the de novo production of ethylene glycol from glucose.** *Metab Eng* 2016, **33:**12-18.

26. Chomvong K, Bauer S, Benjamin DI, Li X, Nomura DK, Cate JH: **Bypassing the Pentose Phosphate Pathway: Towards Modular Utilization of Xylose.** *PLoS ONE* 2016, **11:**e0158111.

27. Deng Y, Mao Y, Zhang X: **Metabolic engineering of E. coli for efficient production of glycolic acid from glucose.** *Biochemical Engineering Journal* 2015, **103:**256-262.

28. Deng Y, Ma N, Zhu K, Mao Y, Wei X, Zhao Y: **Balancing the carbon flux distributions between the TCA cycle and glyoxylate shunt to produce glycolate at high yield and titer in Escherichia coli.** *Metab Eng* 2018, **46:**28-34.

29. Cam Y, Alkim C, Trichez D, Trebosc V, Vax A, Bartolo F, Besse P, Francois JM, Walther T: **Engineering of a Synthetic Metabolic Pathway for the Assimilation of (d)-Xylose into Value-Added Chemicals.** *ACS Synth Biol* 2016, **5:**607-618.

30. Alkim C, Trichez D, Cam Y, Spina L, Francois JM, Walther T: **The synthetic xylulose-1 phosphate pathway increases production of glycolic acid from xylose-rich sugar mixtures.** *Biotechnol Biofuels* 2016, **9:**201.

31. Zahoor A, Otten A, Wendisch VF: **Metabolic engineering of Corynebacterium glutamicum for glycolate production.** *J Biotechnol* 2014, **192 Pt B:**366-375.

32. Bamba T, Yukawa T, Guirimand G, Inokuma K, Sasaki K, Hasunuma T, Kondo A: **Production of 1,2,4-butanetriol from xylose by Saccharomyces cerevisiae through Fe metabolic engineering.** *Metab Eng* 2019, **56:**17-27.

33. Rohles CM, Gläser L, Kohstedt M, Gieβelmann G, Pearson S, Del campo A, Becker J, Wittmann C: **A Bio-based route to the carbon-5 chemical glutaric acid and to bionylon-6,5 using metabolically engineered *Corynebacterium glutamicum*.** *Green Chem* 2018, **20:**4462.

34. Wang J, Wang J, Tai YS, Zhang Q, Bai W, Zhang K: **Rerouting carbon flux for optimized biosynthesis of mesaconate in Escherichia coli.** *Appl Microbiol Biotechnol* 2018, **102:**7377-7388.

35. Pereira B, Li ZJ, De Mey M, Lim CG, Zhang H, Hoeltgen C, Stephanopoulos G: **Efficient utilization of pentoses for bioproduction of the renewable two-carbon compounds ethylene glycol and glycolate.** *Metab Eng* 2016, **34:**80-87.

36. Cam Y, Alkim C, Trichez D, Trebosc V, Vax A, Bartolo F, Besse P, Francois JM, Walther T: **Engineering of a Synthetic Metabolic Pathway for the Assimilation of (d)-Xylose into Value-Added Chemicals.** *ACS Synth Biol* 2015.
